# Subnanometric alkaline-earth oxide clusters for sustainable nitrate to ammonia photosynthesis

**DOI:** 10.1038/s41467-022-28740-8

**Published:** 2022-03-01

**Authors:** Jieyuan Li, Ruimin Chen, Jielin Wang, Ying Zhou, Guidong Yang, Fan Dong

**Affiliations:** 1 Research Center for Environmental and Energy Catalysis, Institute of Fundamental and Frontier Sciences, University of Electronic Science and Technology of China, Chengdu, 611731 China; 2 Yangtze Delta Region Institute (Huzhou), University of Electronic Science and Technology of China, Huzhou, 313000 China; 3 School of New Energy and Materials, Southwest Petroleum University, Chengdu, 610500 China; 4 XJTU-Oxford Joint International Research Laboratory of Catalysis, School of Chemical Engineering and Technology, Xi’an Jiaotong University, Xi’an, 7010049 China

**Keywords:** Pollution remediation, Photocatalysis, Materials for energy and catalysis

## Abstract

The limitation of inert N_2_ molecules with their high dissociation energy has ignited research interests in probing other nitrogen-containing species for ammonia synthesis. Nitrate ions, as an alternative feedstock with high solubility and proton affinity, can be facilely dissociated for sustainable ammonia production. Here we report a nitrate to ammonia photosynthesis route (NO_3_^−^RR) catalyzed by subnanometric alkaline-earth oxide clusters. The catalyst exhibits a high ammonia photosynthesis rate of 11.97 mol g_metal_^−1^ h^−1^ (89.79 mmol g_cat_^−1^ h^−1^) with nearly 100% selectivity. A total ammonia yield of 0.78 mmol within 72 h is achieved, which exhibits a significant advantage in the area of photocatalytic NO_3_^−^RR. The investigation of the molecular-level reaction mechanism reveals that the unique active interface between the subnanometric clusters and TiO_2_ substrate is beneficial for the nitrate activation and dissociation, contributing to efficient and selective nitrate reduction for ammonia production with low energy input. The practical application of NO_3_^−^RR route in simulated wastewater is developed, which demonstrates great potential for its industrial application. These findings are of general knowledge for the functional development of clusters-based catalysts and could open up a path in the exploitation of advanced ammonia synthesis routes with low energy consumption and carbon emission.

## Abstract (web summary)

Photocatalytic reduction of waste nitrate offers an alternative route for ammonia production. Here the authors report BaO clusters on TiO2 for nitrate-ammonia photosynthesis with high ammonia yield.

## Introduction

As an essential chemical, ammonia (NH_3_) is industrially produced via the Haber-Bosch process, which consumes 1.0–2.0% of the world’s energy output and contributes to 1.6% of the world’s carbon emissions^1–4^. As an alternative, artificial electro-/photo-/photoelectrochemical nitrogen reduction reactions (N_2_RRs) for NH_3_ synthesis, inspired by the natural microbial N_2_ fixation, have attracted tremendous research interest^5,6^. Despite great achievements in recent decades, it is inconvenient to overlook that the future of N_2_RRs is plagued by the ultrahigh dissociation energy of the N≡N bond (941 kJ mol^−1^)^7,8^. Inferior catalytic performance is predictable, arising from the limited solubility and low proton affinity of the inert N_2_.

From an energy viewpoint, nitrate ions (NO_3_^−^), as a sustainable N-containing alternative, can be disintegrated at lower dissociation energy of 204 kJ mol^−1^, contributing to an accelerated reaction kinetics for NH_3_ synthesis^9–13^. Besides, the highest valence state of N-element in NO_3_^−^ ensures that the deep reduction reaction can be achieved for selective NH_4_^+^ synthesis. The intermediate-valence N_2_ oxidation and reduction may proceed simultaneously when conducting N_2_RR, which restrains the NH_4_^+^ selectivity^14–16^. Another virtue of using NO_3_^−^ feedstock lies in its rich distribution in wastewater. The abundant nitrate in wastewater offers sufficient reactants for NO_3_^−^ reduction reaction (NO_3_^−^RR) routes^17–20^. Instead of the partial reduction of NO_3_^−^ to N_2_ for its purification, the eight-electron transfer reaction for NO_3_^−^ to NH_4_^+^ synthesis provides an opportunity for the value-added conversion of contaminative NO_3_^−^ into ammonia as an economically competitive product. Also, the wide distribution of general organic matters such as aldehydes and phenols in wastewater is noted, which forms contaminant mixtures with nitrate^21^. These organic matters can serve as the hole sacrificial agents, which accelerates both the NO_3_^−^ reduction for NH_4_^+^ synthesis and pollutants’ oxidation for their degradation. Thus, the development of a NO_3_^−^RR route for NH_4_^+^ production, which provides sustainable N-cycle utilization, has a profound effect in both reducing energy consumption and mitigating environmental anxieties.

Despite its advantages, NO_3_^−^RR also suffers from some inevitable difficulties as an active, yet challenging area of current research. In the eight-electron transfer reaction for NO_3_^−^-NH_4_^+^ synthesis, competitive side reactions may be fierce, mainly ascribed to five-electron transfer for the partial reduction of NO_3_^−^ to N_2_ and hydrogen evolution reaction (HER)^22–24^. Moreover, it is well established that the yield rate and selectivity for NH_4_^+^ dominantly rely on the development of novel catalytic materials, precise regulation of the reaction parameters and systematic investigation of the reaction mechanism. In this scenario, a comprehensive catalysis system requires a rational design to sufficiently promote catalytic performance.

As a typical solid–liquid heterogeneous catalytic reaction, the interaction between catalysts and solvents is essential in the NO_3_^−^RR system. Generally, metal cations (M^x+^) are introduced into solvents, serving as key functional components such as cocatalysts, ionic liquids or electrolytes^25–27^. With the catalytic reaction on stream, the dynamic evolution of M^x+^ can be observed^28,29^. With the assistance of appropriate catalyst support and reaction conditions, the deposition of M^x+^ on a solid surface is expected, which leads to the production of corresponding single atoms, nanoclusters or nanoparticles on a substrate surface^30–32^, thereby modifying the interfacial structure and in situ serving as the catalytic active sites. Key challenge lies in revealing the interfacial structure of active sites for facilitating the ammonia selectivity and suppressing the occurrence of side reactions (HER and NO_3_^−^ to N_2_) to enhance the efficiency.

Here, we demonstrate a general strategy to accomplish the *operando* construction of subnanometric alkaline-earth oxide clusters (MO_NCs_, M=Mg, Ca, Sr or Ba) as the active sites due to the nontoxicity and low price of alkaline-earth metals. Also, the widely investigated TiO_2_ nanosheets (TNS) is applied as the substrate since it is easy to be fabricated and characterized. After the *operando* construction of the BaO_NCs_-TNS composite, a high ammonia photosynthesis rate of 11.97 mol g_metal_^−1^ h^−1^ (89.79 mmol g_cat_^−1^ h^−1^) is reached with nearly 100% selectivity. A total ammonia yield of 0.78 mmol within 72 h is achieved. The local interfacial structure is precisely tailored to strengthen charge transfer at the MO_NCs_/TNS interface. Then, it is revealed that the eight-electron transfer reaction for NO_3_^−^RR is notably accelerated to achieve a high rate for sustainable NH_4_^+^ photosynthesis. The practical application of NO_3_^−^RR route in simulated wastewater is also developed, which establishes an intriguing “sacrificial-agent-free” route for ammonia synthesis and demonstrates great potential for its real industrial application. The current ammonia photosynthesis route could provide an alternative route for nitrogen cycle utilization and promote the development of low-carbon technology.

## Results

### *Operando* construction of the subnanometric clusters

**Fig. 1:**
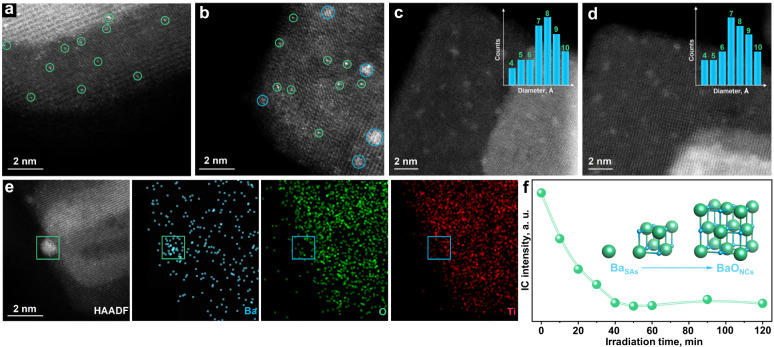
Structure identification of subnanometric BaO nanoclusters (BaO_NCs_) *operando* construction on TiO_2_ nanosheets (TNS) support. **a**–**d** Quasi in situ high-angle annular dark-field scanning transmission electron microscopy (HAADF-STEM) images showing the evolution course from isolated Ba single atoms (Ba_SAs_) to subnanometric (BaO_NCs_) at the irradiation time of 5 min (**a**), 10 min (**b**), 60 min (**c**) and 120 min (**d**) respectively. The related size distribution is labeled as insets (**c**, **d**), in which the range of both *x* (0.4–1.0) and *y* (0–7) axes are set consistently. **e**, HAADF-STEM image (left) and respective elemental mapping images (right) verifying the component of Ba elements on the BaO_NCs_-TNS surface. **f** Variation of Ba^2+^ concentration during the *operando* construction of BaO_NCs_ detected by ion chromatography.

Alkaline-earth ions (50 mg/L) are first injected into the reaction mixture for the NO_3_^−^RR on TNS. Scanning electron microscopy (SEM), transmission electron microscopy (TEM) and X-ray diffraction (XRD) results demonstrate that the morphology and crystal structure of TNS is well maintained after alkaline-earth ion incorporation (Supplementary Figs. 1–3). To reveal the *operando* evolution of the alkaline-earth ions, the mixture is extracted from the reaction on stream. As identified by the quasi in situ high-angle annular dark-field scanning transmission electron microscopy (HAADF-STEM), the *operando* evolution of the alkaline-earth species on the catalyst surface is observed (Fig. 1).

Taking Ba^2+^ as an example, once the BaCl_2_·2H_2_O is introduced into the reaction mixture, single atoms (Ba_SAs_) are constructed on the TNS surface after 5 min of irradiation (Fig. 1a). The subsequent growth and agglomeration of Ba_SAs_ lead to the generation of subnanometric BaO clusters (BaO_NCs_) with a size of ~0.6 nm (Fig. 1b). With prolonged irradiation time (Fig. 1c, d for 60 and 120 min respectively), uniformly dispersed BaO_NCs_ with a mean size of 0.7 ± 0.3 nm are formed on TNS. In addition, the HAADF-STEM elemental mappings (Fig. 1e) confirm that the MO_NCs_ are mainly composed of Ba. It is worth noting that the variation of the Ba^2+^ concentration is revealed by ion chromatography (IC) detection (Fig. 1f). A continuous decrease in the Ba^2+^ concentration is observed within the first 60 min of irradiation. Then, equilibrium is reached to guarantee the subnanometric size of the BaO_NCs_, thereby preventing excessive agglomeration and further growth. In addition, the *operando* construction of MgO_NCs_, CaO_NCs_ and SrO_NCs_ is accomplished under the same procedure as that for the BaO_NCs_ (Supplementary Figs. 4–8), indicating that this is a general strategy to form the MO_NCs_.

### Characterization and electronic properties of the MO_NCs_-TNS composites

**Fig. 2:**
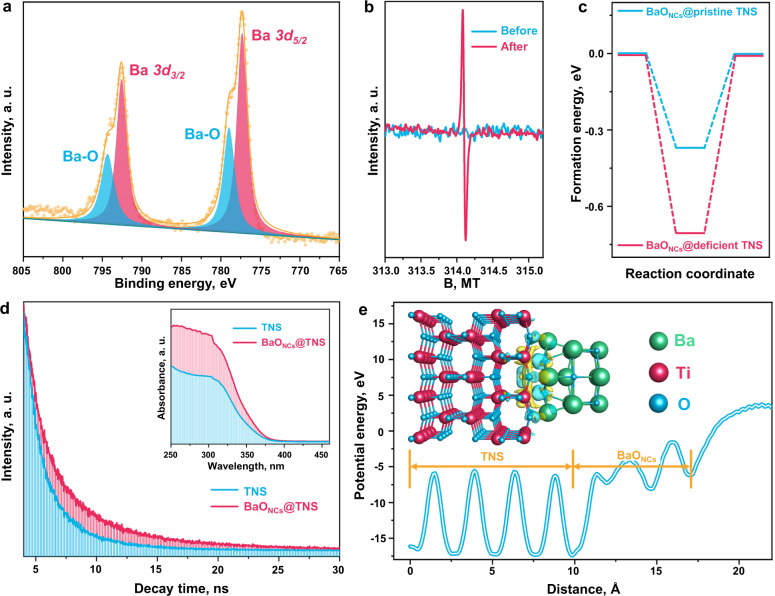
Chemical composition and electronic structure. **a** Ba *3d* X-ray photoelectron spectroscopy (XPS) spectra of BaO_NCs_-TNS. **b** Room temperature solid electron paramagnetic resonance (EPR) results of TNS before and after irradiation for 30 min. **c** Calculated binding energy of BaO_NCs_ deposited at pristine and deficient TNS surfaces respectively. **d** Time-resolved fluorescence emission decay spectra. Inset: UV–vis diffuse reflection spectra (DRS) results. **e** Calculated planer average potential energy profile using Perdew-Burke-Ernzerhof (PBE) + *U* correction. Inset: calculated charge difference distribution at the BaO_NCs_/TNS interface, in which charge accumulation is marked in blue and charge depletion is marked in yellow. The isosurfaces were set to 0.005 eV Å^−3^.

The chemical components and valence states of the BaO_NCs_ were investigated by X-ray photoelectron spectroscopy (XPS, Fig. 2a and Supplementary Fig. 9). The deconvolution of the Ba *3d* XPS spectrum illustrates that the four characteristic peaks are fitted at the binding energies of 794.61, 792.84, 778.99, and 777.15 eV. The peaks located at 792.84 and 777.15 eV correspond to the spin orbits of Ba *3d*_3/2_ and Ba *3d*_*5/2*_ respectively, demonstrating the generation of BaO_NCs_ on the TNS surface. The other two shoulder peaks (794.61 and 778.99 eV) are identified as Ba–O bond formation between the Ba in the BaO_NCs_ and the O in the TNS^33,34^. The concentration of Ba is determined to be 0.75 wt.% and 0.26 at.% by using X-ray fluorescence (XRF) spectroscopy (Supplementary Fig. 10). To investigate the underlying growth pattern and mechanism of BaO_NCs_, electron paramagnetic resonance (EPR) measurements were conducted for the pristine TNS before and after light irradiation (Fig. 2b and Supplementary Fig. 11). The intensified signal for lone-pair electrons after light irradiation discloses that the oxygen vacancies in TNS can be constructed via light irradiation, which agrees with the reported results^35–37^. Furthermore, it is confirmed by density function theory (DFT, Fig. 2c) calculations that the construction of BaO_NCs_ at the defective site of TNS is more energy-favorable (−0.69 eV) than that of pristine TNS (−0.36 V). A uniform pattern for the deposition of other alkaline-earth MO_NCs_ on TNS is confirmed (Supplementary Figs. 12–15), which indicates that the subnanometric MO_NCs_ can be precisely immobilized at the light-induced vacancy sites on TNS with this general method. Since the number of lone-pair electrons is limited at the vacancy sites, the cluster size is restricted at the subnanometric region, which hampers their excessive agglomeration and growth, thus achieving *operando* construction of subnanometric MO_NCs_ at the defective sites of TNS.

Next, the optical and electronic properties of the as-prepared subnanometric MO_NCs_-TNS composites were surveyed. Steady and time-resolved photoluminescence spectra (Fig. 2d and Supplementary Figs. 16 and 17) show that the charge separation capacity of TNS is observably enhanced after the *operando* construction of MO_NCs_. The prolonged carriers’ lifetime is beneficial for the participation of photoinduced electrons to catalyze the NO_3_^–^NH_4_^+^ photosynthesis reaction. Besides, the red shift of the light absorption range (Fig. 2d inset and Supplementary Fig. 18) implies that the light capture and utilization ability is facilitated via the deposition of MO_NCs_. Then the Mott-Schottky spectra and UV–vis diffuse reflection spectra (DRS) were combined to determine the band structures of TNS and BaO_NCs_-TNS (Supplementary Fig. 19). It is noted that the conduction band position of BaO_NCs_-TNS is elevated than that of the pristine TNS, which could enhance the reduction capacity for the elevated NO_3_^−^RR performance. Molecular-level insights into the charge transfer patterns at MO_NCs_/TNS interface were further revealed by DFT calculations (Fig. 2e and Supplementary Figs. 20–22). As supported by both standard Perdew-Burke-Ernzerhof (PBE) functional and PBE + *U* correction calculations for the planar average potential energy profile, a distinct energy gap is generated between the BaO_NCs_ and TNS surface, which facilitates directional electron migration from the BaO_NCs_ to TNS. It is also confirmed by the charge difference distribution (Fig. 2e inset) that intense charge clouds accumulate at the BaO_NCs_/TNS interface, building a unique electronic channel to promote charge transfer. Due to the superior photochemical properties contributed by the *operando* construction of the MO_NCs_-TNS composite, the elevated catalytic performance of the NO_3_^−^RR for NH_4_^+^ photosynthesis is expected.

### NO_3_^−^ to NH_4_^+^ photosynthesis performance

**Fig. 3:**
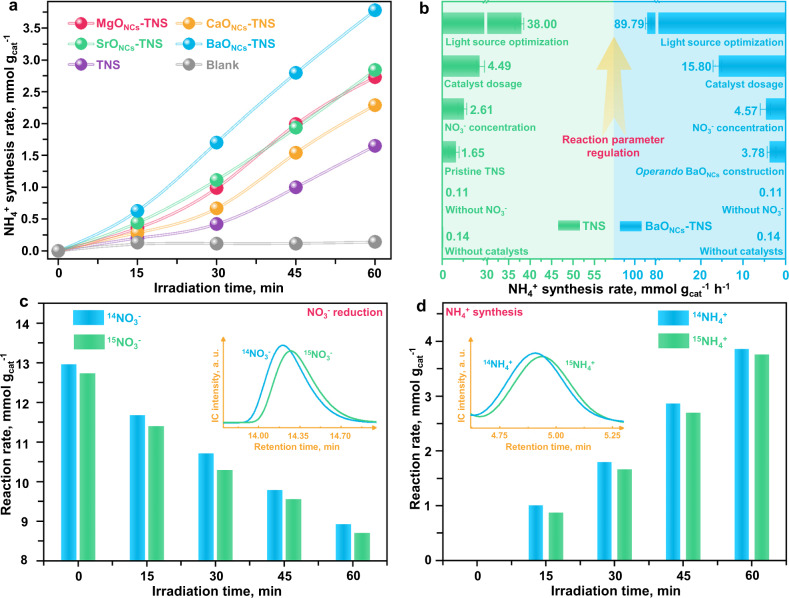
Catalytic performance of NH_3_ photosynthesis on alkaline-earth oxide clusters. **a** Catalytic efficiency tests showing the enhancement of *operando* construction of alkaline-earth oxide clusters on TNS surfaces. **b** Reaction parameter regulation for optimized NH_4_^+^ photosynthesis rates on TNS and BaO_NCs_-TNS respectively. **c**, **d** Quantitative isotope-labeled ^15^NO_3_^−^ study verifying the fed NO_3_^−^ as the source for the prodcuced NH_3_. Inset: raw ion chromatography (IC) spectra for ^14^NO_3_^−^/^15^NO_3_^−^ reduction (**c**) and ^14^NH_4_^+^/^15^NH_4_^+^ generation (**d**) respectively. The *y*-axis of **c**, **d** depict the reaction rates for NO_3_^−^ reduction and NH_4_^+^ production respectively. The inset images are the raw IC data for ^14^NO_3_^−^/^15^NO_3_^−^ (**c**) and ^14^NH_4_^+^/^15^NH_4_^+^ (**d**) respectively. The error bar was drawn based on the calculated standard error of two parallel tests.

The evaluation of the NO_3_^−^RR for NH_4_^+^ photosynthesis was first conducted in 100 mL of KNO_3_ solution (20 mg/L of NO_3_^−^) containing 5.0% ethylene glycol (EG) as the hole sacrificial agent under full-spectrum illumination. Briefly, 5.0 mg of pristine TNS is applied as the catalyst support, in which 50.0 mg/L alkaline-earth ions are injected. As depicted in Fig. 3a, the pristine TNS exhibits nice catalytic activity (1.65 mmol g_cat_^−1^ h^−1^). The oxygen vacancies (OVs) in TNS are identified as the active sites due to the observable OVs construction via light irradiation (Fig. 2b and Supplementary Fig. 11). Most importantly, the *operando* construction of MO_NCs_ (illustrated in Fig. 1f) and enhancement of the NH_4_^+^ photosynthesis rate are simultaneously accomplished with the reaction on stream. Since the construction of MO_NCs_ and NH_4_^+^ synthesis proceed at the same time, the NH_4_^+^ synthesis rate by MO_NCs_ is elevated and the gradual increase of the slope for NH_4_^+^ generation is reasonable. The NH_4_^+^ synthesis rate is increased from 1.65 mmol g_cat_^−1^ h^−1^ with pristine TNS to 3.78 mmol g_cat_^−1^ h^−1^ with BaO_NCs_-TNS, which demonstrates the significant advantage of subnanometric MO_NCs_ as cocatalyst. The apparent quantum efficiency (Supplementary Note 1) for these as-prepared samples is also enhanced in the order of TNS (1.86%) < CaO_NCs_-TNS (2.59%) < MgO_NCs_-TNS (3.09%) < SrO_NCs_-TNS (3.22%) < BaO_NCs_-TNS (3.46%). In addition, the controlled experiment is conducted by adding KCl into the catalysis system of pristine TNS without other cations or anions (Supplementary Fig. 23), which excludes the potential involvement of Cl^−^ from the source of alkaline earth source (MCl_2_·xH_2_O). It is observed that the NO_3_^−^RR to ammonia efficiency is not promoted by the addition of Cl^−^, which identifies that the enhanced activity is contributed by the construction of MO_NCs_-TNS. Since the *operando* production of MO_NCs_ on TNS is preferable to achieve at the OVs sites (Fig. 2c and Supplementary Figs. 12–15), the active sites in MO_NCs_-TNS are regarded as the MO_NCs_@OVs interfaces. Besides, to further unveil the activity origin of MO_NCs_-TNS, we conduct an additional control experiment by replacing the TNS substrate with inert SiO_2_ nanoparticles (Supplementary Fig. 24). It is observed that no NH_4_^+^ can be detected during the simultaneous construction of MO_NCs_-SiO_2_ and NO_3_^−^RR. Hence, it is clarified that the enhanced NH_4_^+^ synthesis efficiency gives credit to the construction of MO_NCs_-TNS composites.

Because the catalytic efficiency is directly related to reaction parameters, the reaction parameters are comprehensively optimized to further promote catalytic performance (Fig. 3b), including the NO_3_^−^ concentration, catalyst dose, and light source. It is observed that the increase in NO_3_^−^ concentration (100.0 mg/L) is beneficial for accelerating the reaction kinetics of NO_3_^−^RR (Supplementary Fig. 25). The saddle point of the catalyst dose is located at 1.0 mg to accomplish the optimal unit activity (Supplementary Fig. 26). Besides, since NO_3_^−^ could be preactivated by UV light, which tailors the coordination environment of the stable NO_3_^−^ and drives it into some active intermediates such as monodentate NO_3_^−^, –NO_2_ and NO_2_^−^ (Supplementary Fig. 27), the utilization of different light sources was tested. Notably, the optimal NH_4_^+^ photosynthesis rate is 38.00 mmol g_cat_^−1^ h^−1^ with pristine TNS after regulating the reaction parameters (Supplementary Fig. 28). Moreover, an accelerated rate for NH_4_^+^ photosynthesis is accomplished on BaO_NCs_-TNS (89.79 mmol g_cat_^−1^ h^−1^) with the reaction parameters of 100 mg L^−1^ of NO_3_^−^, 1.0 mg of BaO_NCs_-TNS catalyst, and UV light irradiation. It is worth mentioning that the introduction of UV light not only increases the energy density but also realizes the preactivation of NO_3_^−^, which exceeds that of the full-spectrum (15.80 mmol g_cat_^−1^ h^−1^) and simulated solar light (3.07 mmol g_cat_^−1^ h^−1^). Then, as shown in the XRF results (Supplementary Fig. 10), 0.75 wt.% of Ba element is detected in the BaO_NCs_-TNS composite. Hence, the rate (per Ba metal) is calculated to be 11.97 mol g_Ba_^−1^ h^−1^. The optimal rate catalyzed by BaO_NCs_-TNS manifests advances in comparison with that of the other ammonia synthesis by using alkaline-earth-containing catalysts (Supplementary Table 1).

To exclude the potential contribution of contaminative N-containing species, blank control experiments were subsequently performed (Supplementary Fig. 29) under the same testing procedure as that without catalysts and NO_3_^−^; these experiments confirm that almost no NH_4_^+^ is produced. Most importantly, a quantitative isotope measurement was executed to test the N source for generating NH_4_^+^ by combining IC and nuclear magnetic resonance (NMR) technologies (Fig. 3c, d and Supplementary Figs. 30–32). K^14^NO_3_ and K^15^NO_3_ solutions are used as N sources. Within 60 min of reaction, ^15^NH_4_^+^ is observably detected when ^15^NO_3_^−^ is employed. In addition, comparable rates for ^14^NO_3_^−^/^15^NO_3_^−^ reduction (Fig. 3c) and ^14^NH_4_^+^/^15^NH_4_^+^ production (Fig. 3d) are identified, confirming that the produced ammonia is directly derived from the nitrate feedstock, thus, the contribution of contaminative N-containing species to ammonia can be neglected.

**Fig. 4:**
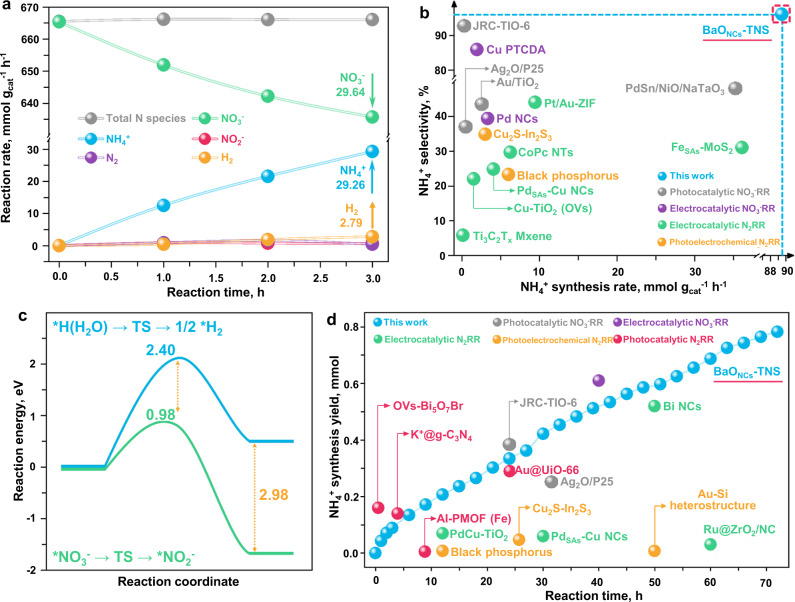
Selectivity and long-term stability tests. **a** NH_4_^+^ selectivity test from NO_3_^−^RR versus the other potential products. The related stand curves were provided in Supplementary Figs. 37–39. **b** Comparison of NH_4_^+^ production rate and selectivity with different ammonia synthesis routes under ambient conditions^11,16,24,59–69^. **c** Calculated activation energy for NO_3_^−^RR for NH_4_^+^ synthesis and water splitting for H_2_ generation. **d** Long-term stability of BaO_NCs_-TNS and comparison of total NH_4_^+^ yield with different NH_4_^+^ synthesis routes^11,16,25,42,59,68–75^. The detailed comparison lists of **b** and **d** are provided in Supplementary Table 2.

We further investigated the selectivity of the NO_3_^−^RR for NH_4_^+^ photosynthesis on BaO_NCs_-TNS (Fig. 4a and Supplementary Figs. 33 and 34). After 3 h’ irradiation, 29.64 mmol g_cat_^−1^ NO_3_^−^ is reduced to generate 29.26 mmol g_cat_^−1^ NH_4_^+^. Besides, the amount of total N species remains stable in the reaction mixture throughout the entire reaction, which implies that the five-electron transfer for partial reduction of NO_3_^−^ to N_2_ is effectively impeded.In addition, generated H_2_ is detected (Supplementary Fig. 35) since water splitting is the primary side reaction in our reaction system. Trace amounts (2.79 mmol g_cat_^−1^ for 3 h) of H_2_ are produced. By comparing the eight-electron NO_3_^−^RR and two-electron water splitting reaction, the selectivity for NH_4_^+^ photosynthesis is determined to reach as high as 97.67%. The NH_4_^+^ synthesis rate and selectivity of the NO_3_^−^RR on BaO_NCs_-TNS are superior among the routes under ambient conditions, exceeding that of the other photocatalytic NO_3_^−^RR works and even some leading electrocatalytic NO_3_^−^RR work (Fig. 4b and Supplementary Table 2). Not surprisingly, the results of this work also exhibit advances in comparison with those of N_2_RR routes at ambient conditions, including electrocatalytic, photocatalytic, and photoelectrochemical methods. Subsequently, the reaction activation energy of NO_3_^−^-NH_4_^+^ synthesis and water splitting for H_2_ production was calculated (Fig. 4c and Supplementary Fig. 36). A distinct activation energy decrease of 1.42 eV is noted for the NO_3_^−^RR compared with that of the water splitting reaction, which can enable efficient inhibition of electron consumption for the side reaction.

Despite the ultra-high NH_4_^+^ photosynthesis rate and selectivity, the total NH_4_^+^ yield is a pivotal benchmark to evaluate the performance of ammonia synthesis catalysts and routes; notably, this benchmark is usually overlooked. Hence, long-term tests were performed to determine the accumulation of NH_4_^+^ (Fig. 4d). It is noted that a small amount of deactivation is observed in the first 3 h, which may be caused by the competing reduction reaction of Ba^2+^ and NO_3_^−^. After the BaO_NCs_ are stably formed on TNS, the significant stable production of NH_4_^+^ on the BaO_NCs_-TNS composite is realized. As a result, a total NH_4_^+^ yield of 0.78 mmol is reached within 72 h. Moreover, the catalyst structure is well maintained after the long-term tests (Supplementary Figs. 40–43). By comparing the NH_4_^+^ synthesis efficiency between the NO_3_^−^RR for NH_4_^+^ photosynthesis and recently reported state-of-the-art routes (Supplementary Table 2), the NH_4_^+^ synthesis rate, selectivity, long-term stability and total NH_4_^+^ yield in this work all demonstrate advances in comparison with those of other ammonia synthesis routes under ambient conditions, including electrocatalytic, photocatalytic and photoelectrochemical methods. In addition, in the area of the NO_3_^−^RR for NH_4_^+^ synthesis, the yield of photosynthesis in this work (0.51 mmol within 40 h) is comparable to that of some leading work using electricity as the catalytic driving force (0.61 mmol within 40 h)^11^. Since the density of energy input for photosynthesis is lower than that of electrocatalysis, the current photosynthesis performance of the NO_3_^−^RR for NH_4_^+^ is very competitive.

### Comprehensive understanding of the reaction mechanism

**Fig. 5:**
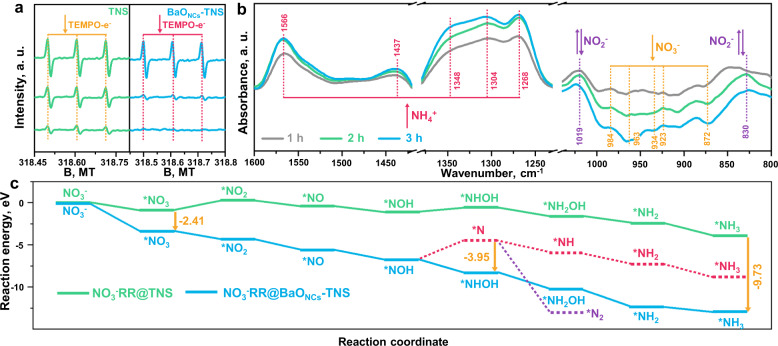
Molecular-level reaction mechanism of NO_3_^−^RR for NH_3_ photosynthesis. **a** EPR spectra for 2,2,6,6-tetramethyl-1-piperidinyloxy (TEMPO)-e^−^ showing the reactive species. **b** In situ diffused reflectance infrared Fourier transform spectroscopy (DRIFTS) revealing the principal reactants and products within the NO_3_^−^RR on BaO_NCs_-TNS. **c** Gibbs free-energy diagram of the reaction coordinates. Steps marked red and purple are the potential side reactions of NO_3_^−^RR on BaO_NCs_-TNS.

To further verify the active radicals responsible for the superior NO_3_^−^RR on the BaO_NCs_-TNS composite, we applied the liquid-state EPR technology using 2,2,6,6-tetramethyl-1-piperidinyloxy (TEMPO) as the trapping reagent (Fig. 5a). The signals of TEMPO decrease rapidly on BaO_NCs_-TNS compared with the TEMPO signals on pristine TNS under light irradiation; this result indicates that more photoexcited electrons are generated and consumed on BaO_NCs_-TNS. The incorporation of H^+^ and TEMPO-e^−^ is significantly strengthened via the *operando* construction of subnanometric BaO_NCs_, generating abundant active protons (H^*^) to catalyze the NO_3_^−^RR for NH_4_^+^ photosynthesis. In addition, enhanced ^•^OH, ^•^O_2_^−^ and ^1^O_2_ production is also observed on BaO_NCs_-TNS (Supplementary Fig. 44). These reactive oxygen species (ROS) are beneficial for the photocatalytic oxidation of EG, which facilitates hole consumption and charge separation.

Moreover, an in situ diffused reflectance infrared Fourier transform spectroscopy (DRIFTS) technique was introduced to dynamically detect the primary reaction process of the NO_3_^−^RR. As shown in Fig. 4b, the characteristic peaks regarding adsorbed NO_3_^−^ are clearly identified at 872, 923, 934, 969, and 984 cm^−1^ ^38,39^. It is noted that the intensity of NO_3_^−^ gradually weakens with prolonged irradiation time, which confirms the consumption and reduction of NO_3_^−^. As the NO_3_^−^RR proceeds, the IR signals of the intermediate NO_2_^−^ are observed at 830 and 1019 cm^−1^ ^40,41^ and increase within the first two hours. After that, a decrease in the NO_2_^−^ signals are noted (blue line in Fig. 4b), which illustrates that more NO_2_^−^ has been consumed than generated. Most importantly, the continuous production of NH_4_^+^ is verified (1268, 1304, 1348, 1437, and 1566 cm^−1^)^42–44^. These results clarify that the NO_3_^−^RR route for NH_4_^+^ photosynthesis is feasible, in which NO_2_^−^ is identified as the principal intermediate product. Based on the in situ DRIFTS detection, the primary reaction pathways of the NO_3_^−^RR for NH_4_^+^ photosynthesis are summarized (Note 1).

**Note 1** Primary reaction pathways of NO_3_^−^ reduction for NH_4_^+^ photosynthesis

1 $${{{{{{\rm{NO}}}}}}}_{3}^{-}+{{{{{{\rm{H}}}}}}}^+\to {}^{\ast }{{{{{\rm{N}}}}}}{{{{{{\rm{O}}}}}}}_{3}+{}^{\ast }{{{{{\rm{H}}}}}}$$

2 $${}^{\ast }{{{{{\rm{N}}}}}}{{{{{{\rm{O}}}}}}}_{3}+{}^{\bullet }{{{{{\rm{H}}}}}}\to {}^{\ast }{{{{{\rm{N}}}}}}{{{{{{\rm{O}}}}}}}_{2}+{}^{\ast }{{{{{\rm{O}}}}}}{{{{{\rm{H}}}}}}$$

3 $${}^{\ast }{{{{{\rm{N}}}}}}{{{{{{\rm{O}}}}}}}_{2}+{}^{\bullet }{{{{{\rm{H}}}}}}\to {}^{\ast }{{{{{\rm{N}}}}}}{{{{{\rm{O}}}}}}+{}^{\ast }{{{{{\rm{O}}}}}}{{{{{\rm{H}}}}}}$$

4 $${}^{\ast }{{{{{\rm{N}}}}}}{{{{{\rm{O}}}}}}+{}^{\bullet }{{{{{\rm{H}}}}}}\to {}^{\ast }{{{{{\rm{N}}}}}}{{{{{\rm{OH}}}}}}$$

5 $${}^{\ast }{{{{{\rm{N}}}}}}{{{{{\rm{OH}}}}}}+{}^{\bullet }{{{{{\rm{H}}}}}}\to {}^{\ast }{{{{{\rm{N}}}}}}{{{{{\rm{HOH}}}}}}$$

6 $${}^{\ast }{{{{{\rm{N}}}}}}{{{{{\rm{HOH}}}}}}+{}^{\bullet }{{{{{\rm{H}}}}}}\to {}^{\ast }{{{{{\rm{N}}}}}}{{{{{{\rm{H}}}}}}}_{2}{{{{{\rm{OH}}}}}}$$

7 $${}^{\ast }{{{{{\rm{N}}}}}}{{{{{{\rm{H}}}}}}}_{2}{{{{{\rm{OH}}}}}}+{}^{\bullet }{{{{{\rm{H}}}}}}\to {}^{\ast }{{{{{\rm{N}}}}}}{{{{{{\rm{H}}}}}}}_{2}+{{{{{{\rm{H}}}}}}}_{2}{{{{{\rm{O}}}}}}$$

8 $${}^{\ast }{{{{{\rm{N}}}}}}{{{{{{\rm{H}}}}}}}_{2}+{}^{\bullet }{{{{{\rm{H}}}}}}\to {}^{\ast }{{{{{\rm{N}}}}}}{{{{{{\rm{H}}}}}}}_{3}$$

The activation and reduction of NO_3_^−^ on the catalyst surface were subsequently revealed by DFT calculations to support the experimental results. As depicted in Supplementary Fig. 45, the adsorption energy and received electrons of NO_3_^−^ at the BaO_NCs_/TNS interface is observably elevated compared with that of pristine TNS, which could promote the NO_3_^−^ reduction process. Furthermore, the Gibbs free-energy diagrams (Δ*G*) were obtained to verify the effect of subnanometric BaO_NCs_ construction on the reaction energy and pathways (Fig. 5c). Referring to the experimental results, we first compared the eight-electron transfer reaction for the synthesis of NO_3_^−^–NH_3_ on pristine TNS (green line in Fig. 5c and Supplementary Fig. 46) and BaO_NCs_-TNS (blue line and Supplementary Fig. 47). It is clearly revealed that facile NO_3_^−^ dissociation (^*^NO_3_–^*^NO_2_) can be accomplished on BaO_NCs_-TNS with an observable decrease in energy compared to that of pristine TNS, which is the dominant reason for the elevated NO_3_^−^RR performance on BaO_NCs_-TNS. As the NO_3_^−^RR proceeds, a total energy decrease of 9.73 eV is noted for efficient NH_3_ synthesis. Two primary competing reaction pathways regarding N_2_ and NH_4_^+^ production were compared (Supplementary Note 2 and 3). A lower ΔG is required for ^*^NOH-^*^NHOH (−1.63 eV) reduction than for ^*^NOH-^*^N (+2.31 eV) reduction (red line in Fig. 5c and Supplementary Fig. 48). Since ^*^N generation is prevented, N_2_ production (purple line in Fig. 5c and Supplementary Fig. 49) is not an optional process on BaO_NCs_-TNS. Besides, the lower activation energy for NO_3_^−^ dissociation (0.98 eV, Fig. 4c) is identified in comparison with that of the water splitting reaction (2.40 eV). Hence, among the three potential products in these reaction pathways (NH_4_^+^, N_2_ and H_2_), the highly selective eight-electron reduction of NO_3_^−^ for NH_4_^+^ photosynthesis is achieved via the assistance of MO_NCs_.

### Practical applications of NO_3_^−^RR in simulated wastewater

**Fig. 6:**
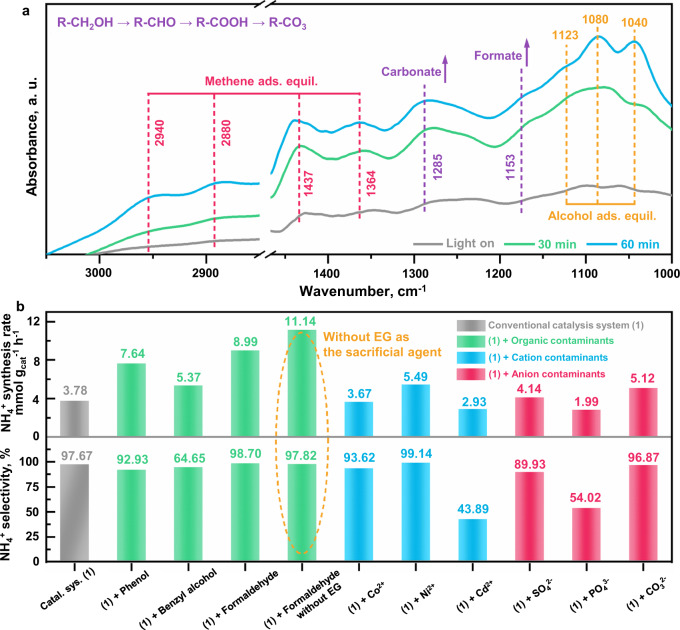
Practical application of NO_3_^−^RR to NH_4_^+^ route in simulated wastewater. **a** In situ DRIFTS for ethylene glycol (EG, hole sacrificial agent) oxidation during the NO_3_^−^RR. **b** Ammonia synthesis rates and selectivity evaluation by adding different types of simulated wastewater into the catalysis system (Catal. Sys.), including the organic matter (phenol, benzyl alcohol and formaldehyde), cation (Co^2+^, Ni^2+^ and Cd^2+^) and anion (SO_4_^2−^, PO_4_^3−^ and CO_3_^2−^) contaminants correspondingly. As for the condition of formaldehyde, the catalytic tests were conducted with and without EG respectively.

The practical application of the as-proposed NO_3_^−^RR for ammonia photosynthesis route was developed. Since the organic matter of EG is applied in the catalysis system (Catal. Sys.) as the hole sacrificial agent, the conversion pathways of EG are investigated via the in situ DRIFTS technology (Fig. 6a). It is observed that the dynamic adsorption equilibrium (Ads. Equil.) of EG is gradually formed based on the detection of methane (2940, 2880, 1437, and 1364 cm^−1^)^45,46^ and alcohol (1123, 1080, and 1040 cm^−1^)^45,47^ species. Then the generation and accumulation of formate (1153 cm^−1^)^48^ and carbonate (1285 cm^−1^)^46,47^ are observed, which can be attributed to the primary products for EG oxidation. Hence, it is concluded that the reactions of EG oxidation and NO_3_^−^ reduction proceed simultaneously, in which the hole consumption by EG oxidation could in turn accelerate the NO_3_^−^RR to promote ammonia synthesis.

Most importantly, it should be noted that abundant organic contaminants are distributed in many NO_3_^−^-containing emission conditions such as agricultural and chemical wastewater degradation and drinking water purification, in which the organic contaminants can be utilized as what is called the hole sacrificial agent. Based on this consideration, phenol, benzyl alcohol and formaldehyde were added into the catal. sys. as potential contaminants respectively (Fig. 6b and Supplementary Fig. 50a–f). It is found that the ammonia synthesis rates and selectivity are all retained, which indicates that the NO_3_^−^RR route is established in the simulated organic wastewater. Interestingly, the ammonia synthesis rates are increased in the order of conventional catal. sys. (3.78 mmol g^−1^ h^−1^) < benzyl alcohol (5.37 mmol g^−1^ h^−1^) < phenol (7.64 mmol g^−1^ h^−1^) < formaldehyde (8.99 mmol g^−1^ h^−1^). It is deduced that the hole consumption capacity of these organic contaminants is higher than that of EG, which leaves more electrons to catalyze the NO_3_^−^RR. Then, the corresponding test of formaldehyde was conducted without EG as the hole sacrificial agent (Fig. 6b and Supplementary Fig. 50g–h). It is observed that the ammonia synthesis rate is further elevated to 11.14 mmol g^−1^ h^−1^ with 97.82% of selectivity noted, which reveals that the formaldehyde can act as the “hole sacrificial agent” more efficiently than that of EG. Based on the organic contaminant investigation in the simulated wastewater, it is clarified that the NO_3_^−^RR route can be developed as a “sacrificial-agent-free” technology for the application of ammonia synthesis in wastewater coupled with the organic pollutants’ oxidation, which demonstrates scientific significance in both areas of environmental remediation and energy conversion. Besides, the addition of cation contaminants (Co^2+^, Ni^2+^, and Cd^2+^, Supplementary Fig. 51) and anion contaminants (SO_4_^2+^, PO_4_^3−^ and CO_3_^2−^, Supplementary Fig. 52) have also been considered, in which the catalytic efficiency is accomplished in general. Some discrepancy in the performance is noted among these ions, which could be raised by the complicated impact of added ions on the catal. sys. and requires further investigation in the future.

## Discussion

In summary, a highly active and selective NO_3_^−^RR for NH_4_^+^ photosynthesis was achieved by *operando* construction of subnanometric MO_NCs_ on TNS. The dynamic evolution pattern, growth mechanism, and interfacial structure of MO_NCs_ were characterized and well-defined. A superior NH_4_^+^ photosynthesis rate, selectivity, long-term stability and total yield were achieved among the various NH_4_^+^ synthesis routes under ambient conditions. Then it was proposed that the unique electronic structure at the MO_NCs_/TNS interface was mainly responsible for the enhanced NO_3_^−^ dissociation and eight-electron reduction reaction. The practical application of NO_3_^−^RR route in simulated wastewater was developed, which demonstrated scientific significance in both areas of environmental remediation and energy conversion. The discovery of subnanometric MO_NCs_ for sustainable NO_3_^−^-NH_4_^+^ photosynthesis is inspiring and is of general knowledge, thereby providing numerous opportunities for research into cluster chemistry and artificial photosynthesis.

## Methods

### Chemicals

All chemicals were purchased without further treatment. The respective source and purity were listed in Supplementary Table 3.

### Synthesis of TNS

The synthesis of TiO_2_ nanosheets (TNS) was conducted via slight modification of the reported work^49,50^. In a typical preparation procedure, 3 mL of HF solution was dropped slowly into 25 mL of Ti(OBu)_4_•(TBOT) under continuous magnetic stirring for 2 h until the solution formed into a gel-like solution. Then the mixture was transferred into a Teflon autoclave with a volume of 50 mL and then heated at 180 °C for 24 h. After naturally cooling down to room temperature, the powder was separated and collected by high-speed centrifugation at 10,000 rpm for 5 min with distilled water (DI) and ethanol washing for at least 10 times respectively, which removes the residual organic matter and F^−^. At last, the obtained sample was dried at 80 °C overnight in the vacuum drying oven.

### Construction of MO_NCs_ on TNS

Five milligrams of TNS powder was dispersed in 100 mL of reaction solution containing 10 mg/L of NO_3_^−^-N, 50 mg/L of alkaline earth chlorides (MgCl_2_, CaCl_2_, SrCl_2_, and BaCl_2_ respectively) and 10 mL EG. Then the mixture was transferred into a photocatalytic reactor (MC-GF250, Merry Change, China) and degassed for soluble air with high-purity Ar (99.999%) at 50 mL/min for 30 min under stirring. A 300 W Xe lamp (MC-X301B, Merry Change, China) was used at the light source. After 1 h’s irradiation with continuous Ar pumping and stirring, the obtained powder was collected by washing with ethanol and DI for three times respectively. With the irradiation on stream, the reaction mixture was extracted several times for the tests of M^X+^ concentration and *operando* evolution of MO_NCs_ on the substrate surface.

### Catalyst characterization

The crystal information was examined by the X-ray diffraction (XRD, model D/max RA, Rigaku Co.) technology. The morphology was surveyed by scanning electron microscopy (SEM, XL30 ESEM FEG), transmission electron microscopy (TEM, FEI Talos F200S). The *operando* evolution of MO_NCs_ was verified by quasi in situ high-angle annular dark-field scanning transmission electron microscopy (HAADF-STEM, JEOL JEM-ARM200F) with spherical aberration correction to investigate the morphology at the atomic scale. The chemical composition was tested via the X-ray photoelectron spectroscopy (XPS, Thermo Scientific K-Alpha plus) with an Al Kα X-ray light source. The elemental component analysis was conducted by X-ray fluorescence (XRF, BRUKER, M4 TORNADO). The solid-state EPR (JES FA200) spectra were performed to identify the vacancy signals.

### Optical and electronic property identification

The light absorption capacity was performed by a scanning UV–vis spectrophotometer (Shimadzu UV-2450) outfitted with an integrating sphere, using barium sulfate as the comparison sample. The Mott-Schottky spectra were conducted using catalysts/C, Ag/AgCl and Pt as working, reference and counter electrodes respectively on an electrochemical workstation (CHI-660E), and the results were recorded from −1.0 to 1.5 V at 1000 Hz without light irradiation. The steady photoluminescence (PL) spectra were investigated using a fluorescence spectrophotometer (Edinburgh Instruments FLSP-920). Time-resolved fluorescence emission decay spectra (PicoQuant Fluotime 300) were carried out to verify the carrier’s life time under light irradiation. The liquid-state EPR spectra of active radicals were obtained on a JES FA200 spectrometer to investigate the production of the ROS under light illumination. The 5, 5-Dimethyl-1-Pyrroline-N-Oxide (DMPO) was used as the trapping agent to confirm the involvement of DMPO-^•^OH and DMPO-^•^O_2_^−^ respectively in aqueous methanol dispersion. 4-oxo-2, 2, 6, 6-Tetramethyl-1-Piperidinyloxy (TEMP) was applied to survey the TEMP-^1^O_2_ and TEMP-1-oxyl (TEMPO) was used to characterize the photo-induced electrons (TEMPO-e^−^).

### DFT calculation

The spin-polarized DFT calculations were operated with the “Vienna ab initio simulation package” (VASP 5.4), in which the PBE exchange-correlation functional was included^51–53^. The PBE + *U* correction (*U* = 4.0 eV) was implemented to account for the on-site charge interaction of the d electrons in Ti elements^54^, which improved the accuracy for the calculations of electron migration at the MO_NCs_-TNS interfaces. The cut-off energy was set to 400 eV. K points in the Brillouin zone were set to 5 × 5 × 1 for both structural and electronic optimization. Geometry relaxation was achieved after the residual forces were smaller than 0.01 eV Å^−1^. The Gaussian smearing width was set to 0.2 eV. The initial calculation model of TNS contains 60 Ti atoms and 120 O atoms respectively (Supplementary Fig. 12a). The typical [001] facet is exposed for further calculations. The lattice parameters were set to 11 × 15 × 25 Å^3^, which contains a vacuum slab of 15 Å to impede the potential interaction between neighboring lattices. The initial calculation models of MgO_NCs_, CaO_NCs_, SrO_NCs_ and BaO_NCs_ were constructed with the cluster size of 0.73, 0.83, 0.89, and 0.96 nm respectively (Supplementary Fig. 12b–e)

The adsorption energy (*E*_ads_) for molecules was calculated as follows:

9 $${E}_{{{{{{\rm{ads}}}}}}}={E}_{{{{{{\rm{tot}}}}}}}-{E}_{{{{{{\rm{cat}}}}}}.}-{E}_{{{{{{\rm{mol}}}}}}}$$

where *E*_tot_, *E*_cat._, and *E*_mol_ depicted the total energy of adsorption structure, catalyst support, and isolated molecule respectively.

The Gibbs free energy variation (Δ*G*)^55,56^ between the initial state (IS) and final state (FS) was determined with the following equation

10 $$\Delta G={E}_{{{{{{\rm{FS}}}}}}}-{E}_{{{{{{\rm{IS}}}}}}}+\Delta {E}_{{{{{{\rm{ZPE}}}}}}}-T\Delta S$$

where *E*_FS_ and *E*_IS_ referred to the DFT total energy for FS and IS correspondingly. Δ*E*_ZPE_ and Δ*S* denoted the variation of zero-point energy and entropy. The room temperature (*T*, 298.15 K) was applied.

The climbing image-nudged elastic band (CI-NEB)^57,58^ code was conducted to identify the reaction coordinates from IS to FS, which located the transition state (TS) with single imaginary frequency verification. The activation energy (*E*_a_) and reaction energy (*E*_r_) were defined as follows

11 $${E}_{{{{{{\rm{a}}}}}}}={E}_{{{{{{\rm{TS}}}}}}}-{E}_{{{{{{\rm{IS}}}}}}}$$

12 $${E}_{{{{{{\rm{r}}}}}}}={E}_{{{{{{\rm{FS}}}}}}}-{E}_{{{{{{\rm{IS}}}}}}}$$

where *E*_IS_, *E*_TS_, and *E*_FS_ were DFT calculated total energy of IS, TS, and FS respectively.

### NO_3_^−^–NH_4_^+^ photosynthesis efficiency test

Photosynthesis tests were first conducted to determine the activity enhancement by operando MO_NCs_ construction. In a typical experimental procedure, 5 mg of TNS powder was dispersed in 100 mL of reaction solution containing 10 mg/L of NO_3_^−^-N, 50 mg/L of alkaline earth chlorides (MgCl_2_, CaCl_2_, SrCl_2_, and BaCl_2_ respectively) and 10 mL EG. Then the mixture was transferred into a photocatalytic reactor (MC-GF250, Merry Change, China) and degassed for soluble air with Ar at 50 mL/min for 30 min under stirring. A 300 W Xe lamp (MC-X301B, Merry Change, China) was used at the light source. After the photocatalysis reaction, the *operando* construction of MO_NCs_ on TNS is accomplished. The photocatalysts were collected and washed for further characterization. The blank control experiment was also performed, which excluded the catalysts and NO_3_^−^-N respectively. The consumed NO_3_^−^ and produced NH_4_^+^ were both detected by ion chromatography (IC, Shimadzu IC-16 for NH_4_^+^ and Analysis Lab CS2000 for NO_3_^−^ respectively). After that, the reaction parameter was comprehensively optimized to obtain the optimal NH_4_^+^ photosynthesis efficiency, which included NO_3_^−^-N concentration (from 10 to 500 mg/L), catalyst dosage (from 0.5 to 5 mg) and the irradiation source (UV, full spectrum and simulated solar light). The pH of this catalytic system remains at ca. 7.0 during the test since the KNO_3_ and EG consist of neutral solutions (Supplementary Fig. 53). The temperature is controlled at 25 °C by using the circulating chiller (Supplementary Fig. 54).

The quantitative ^15^N isotope tracing measurement was conducted, using 10 mg/L of K^14^NO_3_ and K^15^NO_3_ as the feedstock respectively, the produced ^14^NH_4_^+^ and ^15^NH_4_^+^ were quantified by IC. Then the ^1^H NMR (Bruker 400 M) was used to complement the IC results. As for the long-term stability test, the NO_3_^−^-N concentration was increased to 500 mg/L to guarantee sufficient feedstock. Meanwhile, the catalyst dosage was increased to 50 mg to elevate the total yield of NH_4_^+^. In order to investigate the NH_4_^+^ selectivity, the H_2_ was also detected during the NO_3_^−^RR, using gas chromatography (Kechuang GC 2002) with the thermal conductivity detector (TCD). The selectivity was calculated as follows

13 $${{{{{{\rm{NH}}}}}}}_{4}^+\,{{{{{\rm{Selectivity}}}}}}=[8\,{{{{{\rm{Yield}}}}}}\,({{{{{{\rm{NH}}}}}}}_{4}^+)]/[2\,{{{{{\rm{Yield}}}}}}\,({{{{{{\rm{H}}}}}}}_{2})+5\,{{{{{\rm{Yield}}}}}}\,(1/2\,{{{{{{\rm{N}}}}}}}_{2})+8\,{{{{{\rm{Yield}}}}}}\,({{{{{{\rm{NH}}}}}}}_{4}^+)]\,\times 100 \%$$

### In situ DRIFTS investigation

In situ diffuse reflectance infrared Fourier transform spectroscopy (DRIFTS, Bruker INVENIO R) was utilized to monitor the adsorbed species on the BaO_NCs_-TNS surface within the reaction process, in which an in situ diffuse-reflectance cell (Harrick) and a reaction chamber (HVC) were equipped. Before measurement, the catalyst was mixed into 100 mg/L of NO_3_^−^-N solution by continuous stirring and then dried at 110 °C in a vacuum drying oven. The as-prepared sample was transferred in the reaction chamber and heated for 30 min at 110 °C to completely remove the adsorbed species on the surface. High-purity He (99.999%) was continuously pumped into the reaction system to maintain the inert atmosphere. A Xe lamp was used as the light source. The IR detection was conducted during the light irradiation.

### Practical applications in simulated wastewater

Some potential contaminants were added into the catalysis system of BaO_NCs_-TNS to evaluate the NO_3_^−^RR performance in simulated wastewater, including phenol, benzyl alcohol, and formaldehyde as organic matters, Co^2+^, Ni^2+^, and Cd^2+^ as cations, SO_4_^2−^, PO_4_^3−^ and CO_3_^2−^ as anions respectively. CoCl_2_, NiCl_2_, and CdCl_2_ were used as the source of cations. K_2_SO_4_, K_3_PO_4_, and K_2_CO_3_ were used as the source of anions respectively. Based on the formaldehyde test, the EG is excluded from the reaction mixture for comparison. The concentration variation of SO_4_^2−^, PO_4_^3−^, and CO_3_^2−^ is determined by ion chromatograph (Shimadzu IC-16). An inductive coupled plasma emission spectrometer (Agilent ICPOES730) is applied to reveal the concentration of Co^2+^, Ni^2+^, and Cd^2+^. Phenol and benzyl alcohol were tested on the liquid chromatography (Shimadzu LC-20AT). In addition, we measured the concentration of formic acid as the oxidative product of formaldehyde by IC (Shimadzu IC-16).

## Supplementary information

Supplementary Information

Peer review file

Description of Additional Supplementary Files

Supplementary Dataset 1

Supplementary Dataset 2

## Data Availability

All data generated in this study are provided in the Source Data files, in which the data presented in Figures from the Maintext and Supplementary Information are listed in the Excel files of Source Data 1 and Source Data 2 respectively.

## Code availability

Only the commercial codes were used in this work (See references).
