# Supplementary material for: Subnanometric alkaline-earth oxide clusters for sustainable nitrate to ammonia photosynthesis
Source: Nat Commun. 2022 Mar 1;13:1098. doi: 10.1038/s41467-022-28740-8 (PMC8888631; doi:10.1038/s41467-022-28740-8)
Supplement: Supplementary file 1 — Supplementary Information [file 41467_2022_28740_MOESM1_ESM.pdf]

Supplementary Information

Subnanometric alkaline-earth oxide clusters for sustainable nitrate to ammonia photosynthesis

Jieyuan Li,[1,2] Ruimin Chen,[1] Jielin Wang,[1] Ying Zhou,[3] Guidong Yang,[4] Fan Dong[1,2*]

1 Research Center for Environmental and Energy Catalysis, Institute of Fundamental and Frontier Sciences, University of Electronic Science and Technology of China, Chengdu 611731, China.

2 Yangtze Delta Region Institute (Huzhou), University of Electronic Science and Technology of China, Huzhou 313000, China.

3 School of New Energy and Materials, Southwest Petroleum University, Chengdu 610500, China.

4 XJTU-Oxford Joint International Research Laboratory of Catalysis, School of Chemical Engineering and Technology, Xi'an Jiaotong University, Xi'an 710049, China.

* Corresponding author: Fan Dong (dongfan@uestc.edu.cn; dfctbu@126.com)

Table of Context

Supplementary Figures **S4**
Supplementary Figure 1 XRD patterns S4
Supplementary Figure 2 SEM images S4
Supplementary Figure 3 TEM images S5
Supplementary Figure 4 HAADF-STEM images S5
Supplementary Figure 5 Elemental mappings for MgO_{NCs}-TNS S6
Supplementary Figure 6 Elemental mappings for CaO_{NCs}-TNS S6
Supplementary Figure 7 Elemental mappings for SrO_{NCs}-TNS S7
Supplementary Figure 8 IC data for metal ions S7
Supplementary Figure 9 XPS survey spectra S8
Supplementary Figure 10 XRF results S8
Supplementary Figure 11 Solid-state EPR results S9
Supplementary Figure 12 Relaxed calculation models S10
Supplementary Figure 13 Calculation for MgO_{NCs} deposition S10
Supplementary Figure 14 Calculation for CaO_{NCs} deposition S11
Supplementary Figure 15 Calculation for SrO_{NCs} deposition S11
Supplementary Figure 16 PL spectra S12
Supplementary Figure 17 Time-resolved fluorescence spectra S12
Supplementary Figure 18 UV-vis DRS results S13
Supplementary Figure 19 Estimated band structures S13
Supplementary Figure 20 Calculated charge properties for MgO_{NCs}-TNS S14
Supplementary Figure 21 Calculated charge properties for CaO_{NCs}-TNS S15
Supplementary Figure 22 Calculated charge properties for SrO_{NCs}-TNS S15
Supplementary Figure 23 Control experiment by adding Cl^- S16
Supplementary Figure 24 Control experiment by inert SiO_2 .... S16
Supplementary Figure 25 NO_3^- concentration optimization S17
Supplementary Figure 26 Catalyst dosage optimization S17
Supplementary Figure 27 NO_3^- preactivation by UV light S18
Supplementary Figure 28 Light source optimization S19
Supplementary Figure 29 Blank control experiment S20
Supplementary Figure 30 Raw IC data for $^{14}NO_3^-$/$^{14}NO_2^-$ and $^{15}NO_3^-$/$^{15}NO_2^-$ S20
Supplementary Figure 31 Raw IC data for $^{14}NH_4^+$ and $^{15}NH_4^+$ S21
Supplementary Figure 32 ^1H NMR spectra for $^{14}NH_4^+$ and $^{15}NH_4^+$ S21
Supplementary Figure 33 Raw IC data for NO_3^- consumption S22
Supplementary Figure 34 Raw IC data for NH_4^+ generation S22
Supplementary Figure 35 GC signals for H_2 generation S23
Supplementary Figure 36 Reaction coordinates of NO_3^-RR and water splitting .S23
Supplementary Figure 37 Plotting of NH_4^+ standard curves S24

Supplementary Figure 38 Plotting of NO_3^- standard curves S25
Supplementary Figure 39 Plotting of NO_2^- standard curves S25
Supplementary Figure 40 SEM images after reactions S26
Supplementary Figure 41 TEM images after reactions S26
Supplementary Figure 42 HAADF-STEM images after reactions S27
Supplementary Figure 43 XRD patterns after reactions S27
Supplementary Figure 44 Liquid-state EPR results for ROS generation S28
Supplementary Figure 45 Calculated NO_3^- adsorption S29
Supplementary Figure 46 Calculated NO_3^- reduction on TNS S30
Supplementary Figure 47 Calculated NO_3^- reduction on BaO_{NCs}-TNS S31
Supplementary Figure 48 Calculated potential side reactions 1 .... S32
Supplementary Figure 49 Calculated potential side reactions 2 .... S33
Supplementary Figure 50 Efficiency tests in organic wastewater S33
Supplementary Figure 51 Efficiency tests in cation wastewater S34
Supplementary Figure 52 Efficiency tests in anion wastewater S34
Supplementary Figure 53 pH variation during tests S35
Supplementary Figure 54 Temperature variation during tests S35
Supplementary Notes S36
Supplementary Note 1 Apparent quantum yield calculation details S36
Supplementary Note 1 Competing reaction pathways 1 .... S37
Supplementary Note 2 Competing reaction pathways 2 .... S37
Supplementary Tables S38
Supplementary Table 1 Comparison of the alkaline-earth-based catalysts S38
Supplementary Table 2 Comparison of different ammonia synthesis routes S39
Supplementary Table 3 Source and purity of chemicals S44

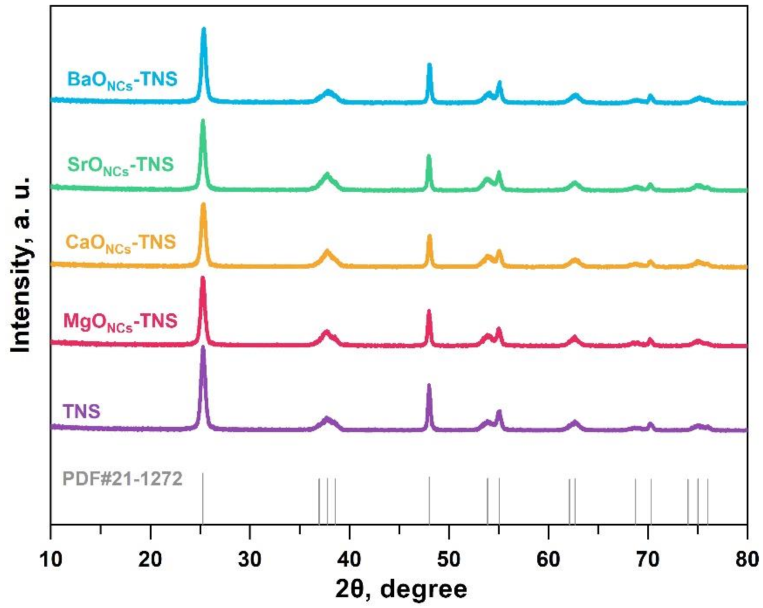

Supplementary Figure 1. XRD patterns for pristine and MO$_{NCs}$ deposited TNS.

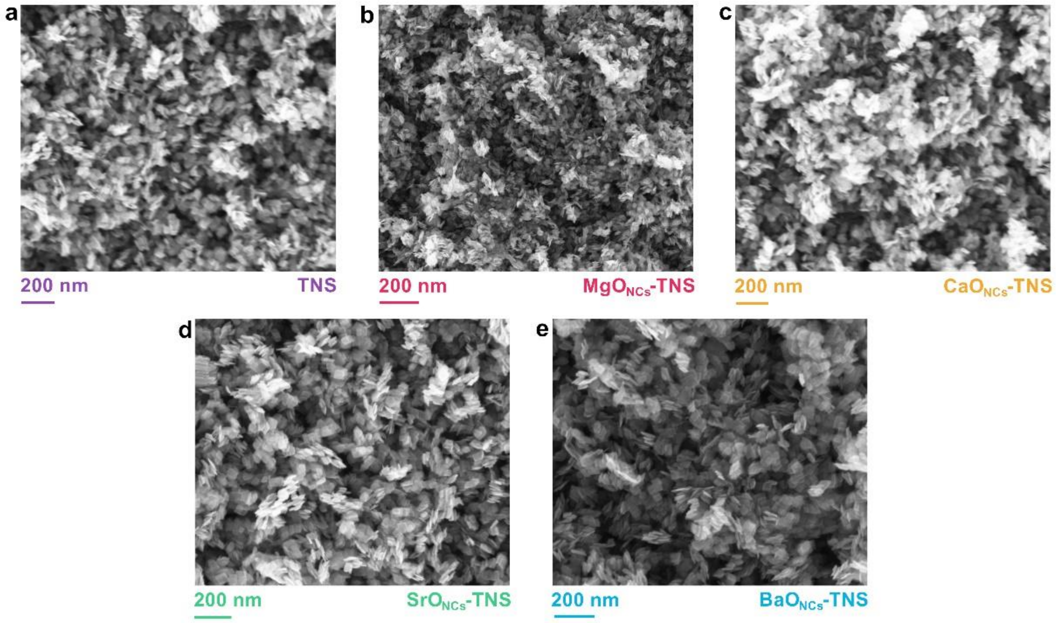

Supplementary Figure 2. SEM images TNS **(a)**, MgO$_{NCs}$-TNS **(b)**, CaO$_{NCs}$-TNS **(c)**, SrO$_{NCs}$-TNS **(d)** and BaO$_{NCs}$-TNS **(e)**.

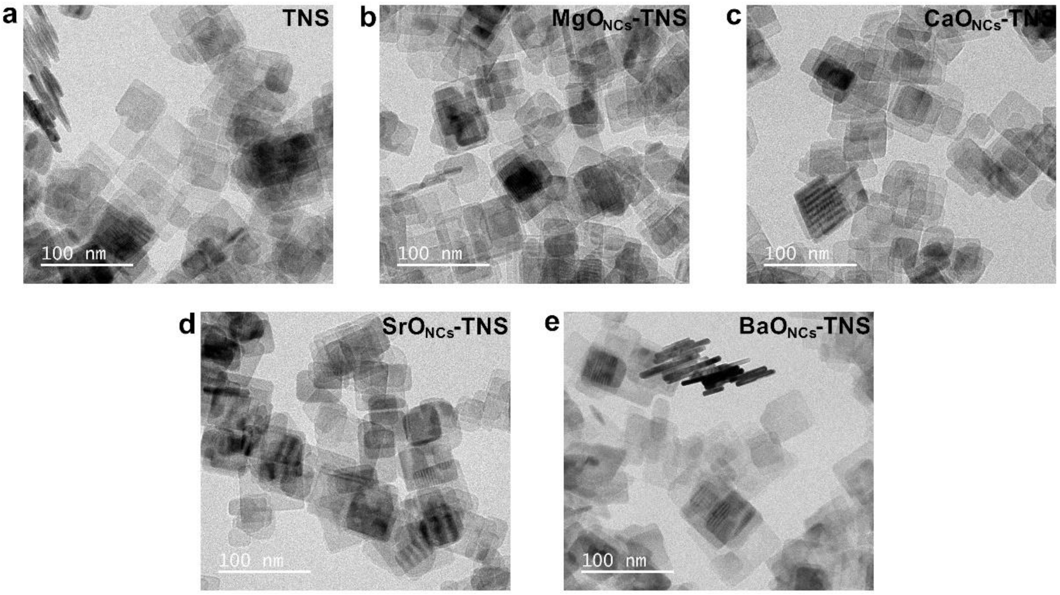

Supplementary Figure 3. TEM images of TNS **(a)**, MgO_{NCs}-TNS **(b)**, CaO_{NCs}-TNS **(c)**, SrO_{NCs}-TNS **(d)** and BaO_{NCs}-TNS **(e)**.

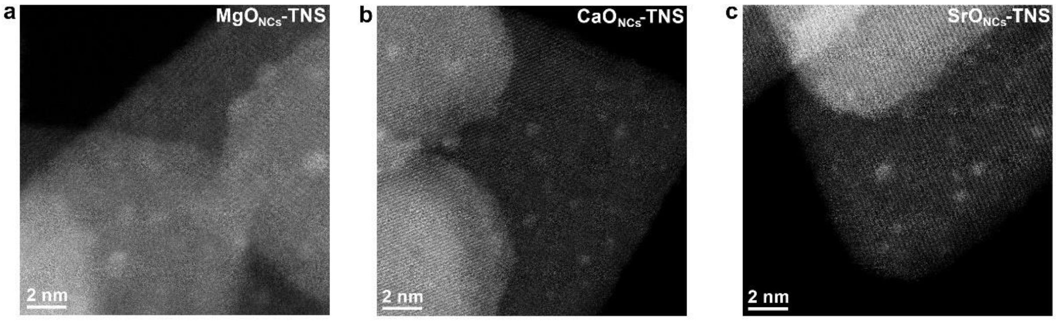

Supplementary Figure 4. HAADF-STEM for MgO_{NCs}-TNS **(a)**, CaO_{NCs}-TNS **(b)**, SrO_{NCs}-TNS **(c)**.

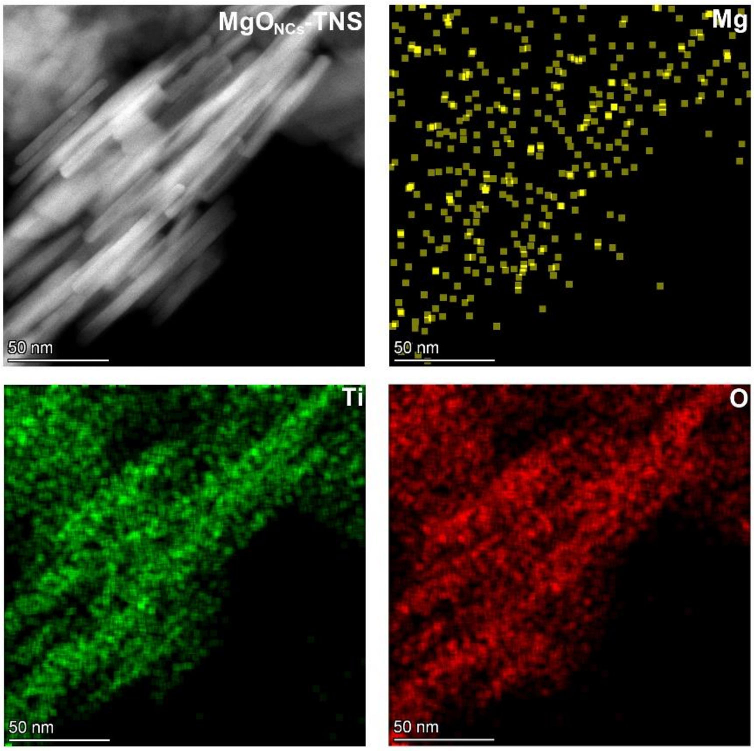

Supplementary Figure 5. TEM and elemental mapping images for MgO_{NCs}-TNS.

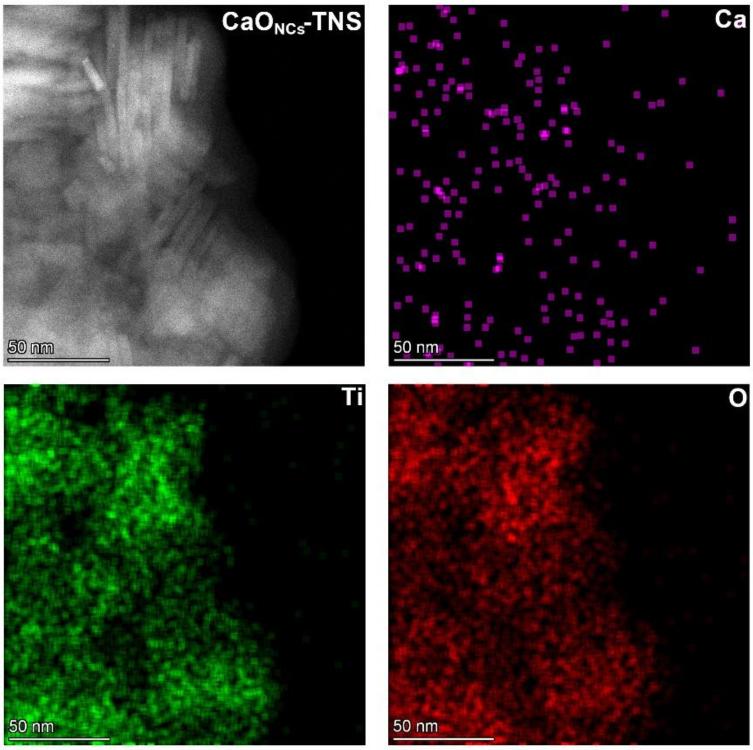

Supplementary Figure 6. TEM and elemental mapping images for CaO_{NCs}-TNS.

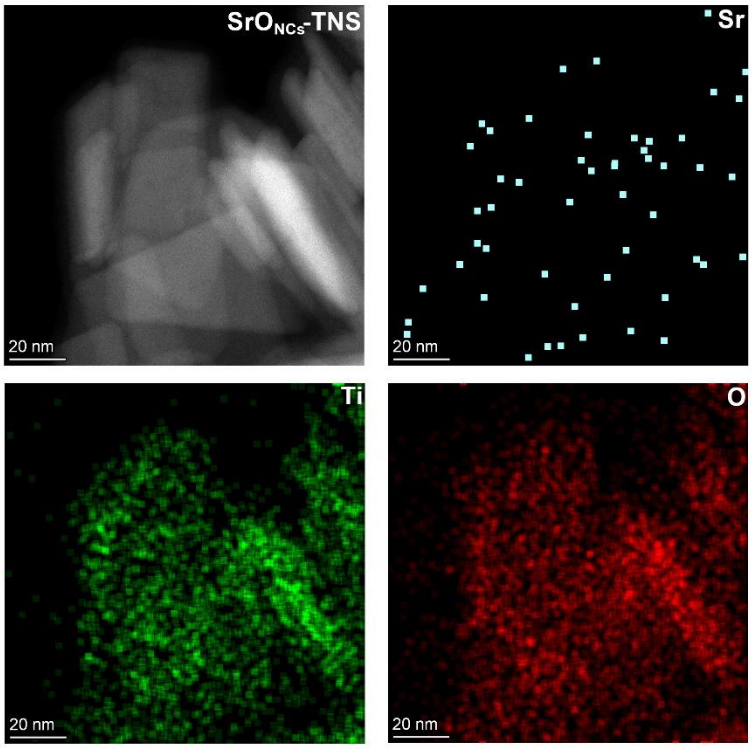

Supplementary Figure 7. TEM and elemental mapping images for SrO$_{NCs}$-TNS.

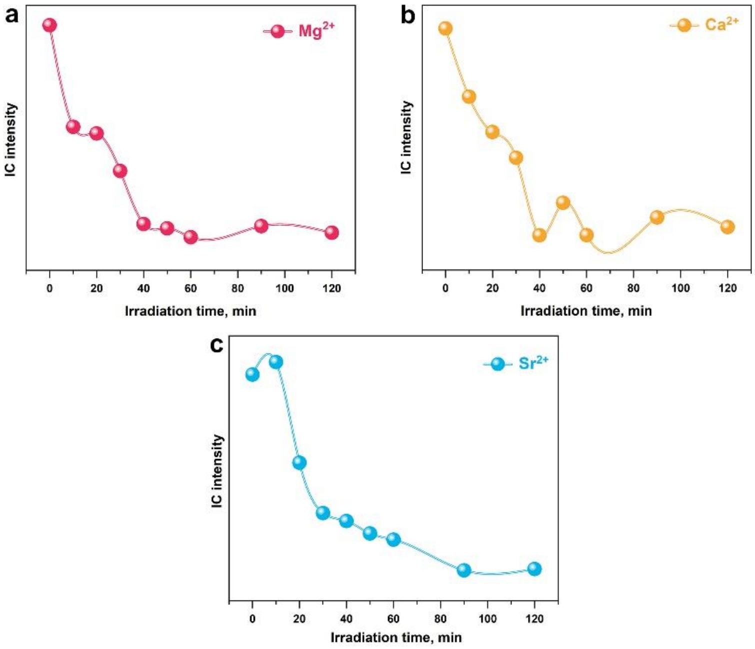

Supplementary Figure 8. Variation of IC signals for Mg^{2+} **(a)**, Ca^{2+} **(b)** and Sr^{2+} **(c)** ions during the operando construction of corresponding MO$_{NCs}$ on TNS.

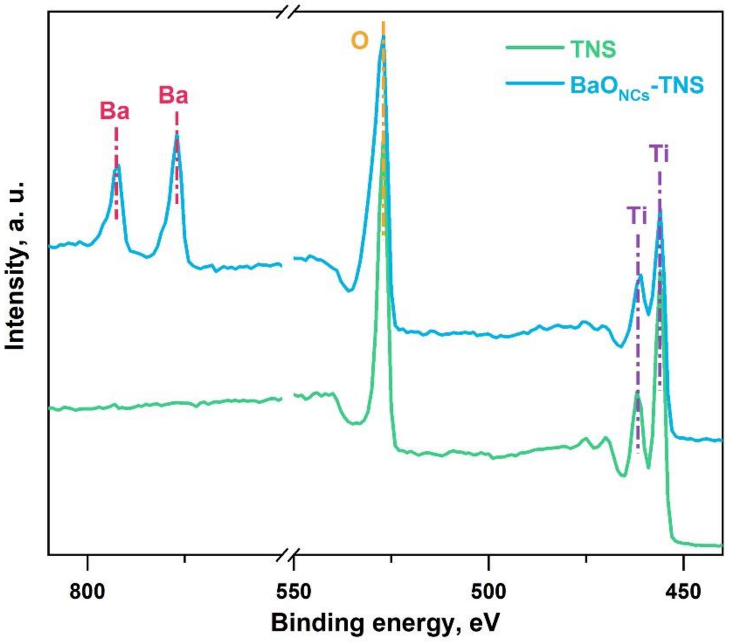

Supplementary Figure 9. XPS survey spectra for pristine and BaO_{NCs} deposited TNS.

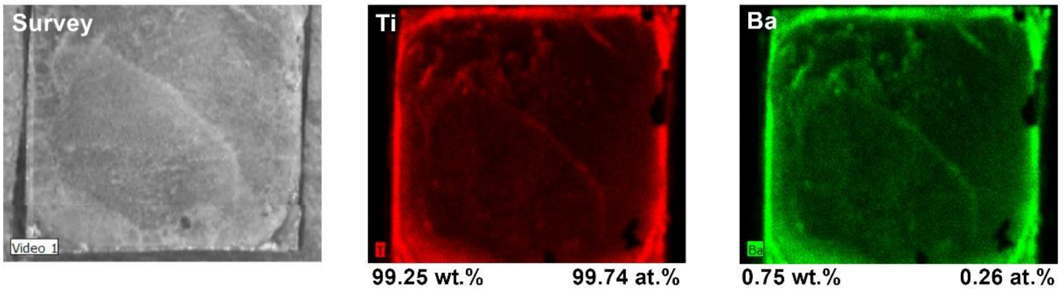

Supplementary Figure 10. XRF results for BaO_{NCs}-TNS.

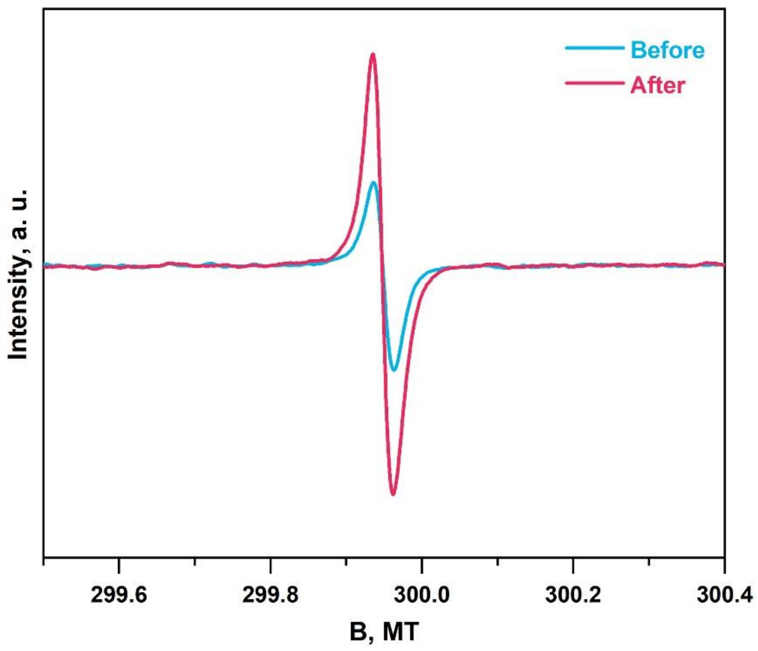

Supplementary Figure 11. Low temperature (120 K) solid-state EPR results of TNS before and after irradiation for 30 min.

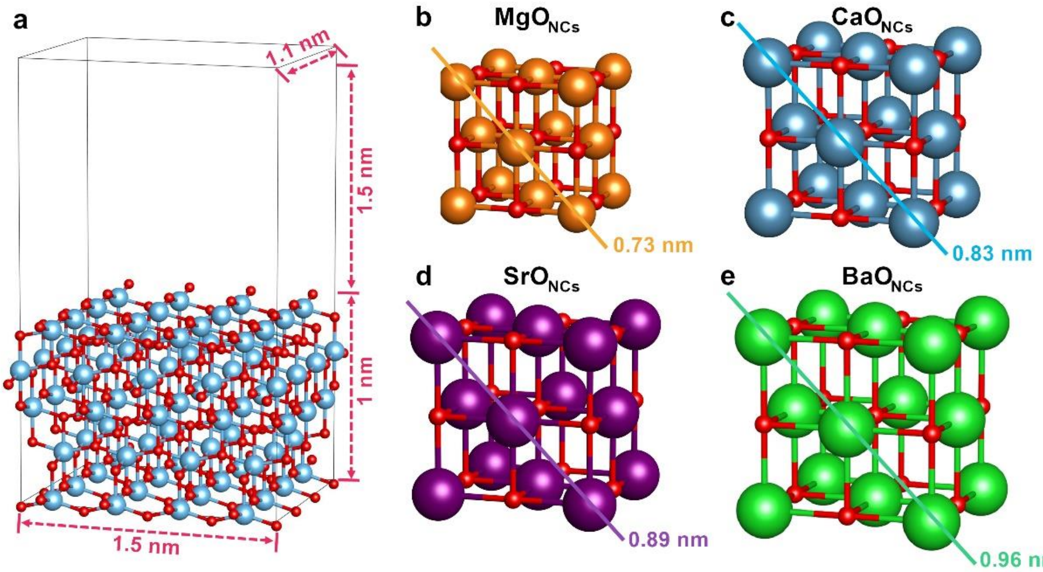

Supplementary Figure 12. Initial calculation models for pristine TNS **(a)**, MgO_{NCs}-TNS **(b)**, CaO_{NCs}-TNS **(c)**, SrO_{NCs}-TNS **(d)** and BaO_{NCs}-TNS **(e)**. The blue, red, orange, dark blue, purple and green spheres depict Ti, O, Mg, Ca, Sr and Ba atoms respectively.

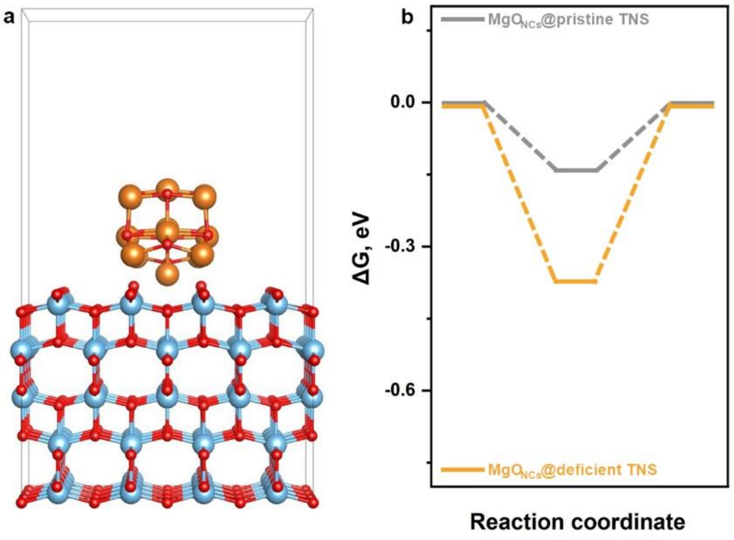

Supplementary Figure 13. a. Relaxed micro-structure of MgO_{NCs} deposited at TNS surface. The blue, red and orange spheres depict Ti, O and Mg atoms respectively. **b.** Calculated binding energy of BaO_{NCs} deposited at pristine and deficient TNS surfaces, respectively.

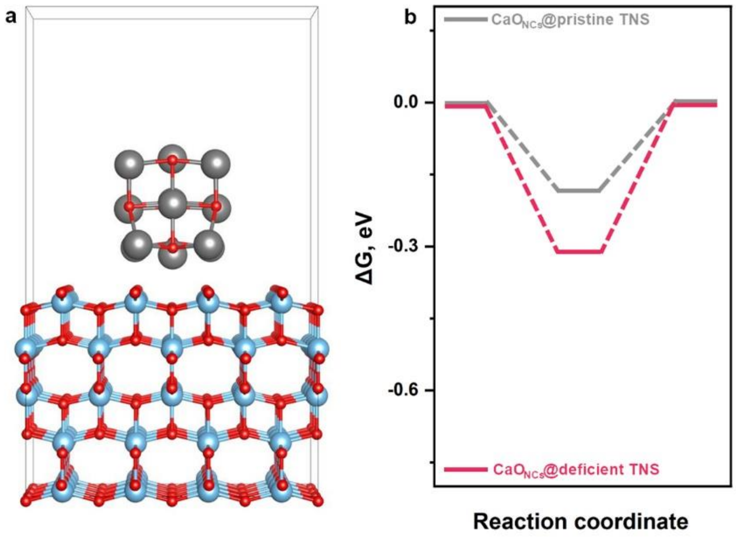

Supplementary Figure 14. a. Relaxed micro-structure of CaO_{NCs} deposited at TNS surface. The blue, red and grey spheres depict Ti, O and Ca atoms respectively. **b.** Calculated binding energy of CaO_{NCs} deposited at pristine and deficient TNS surfaces respectively.

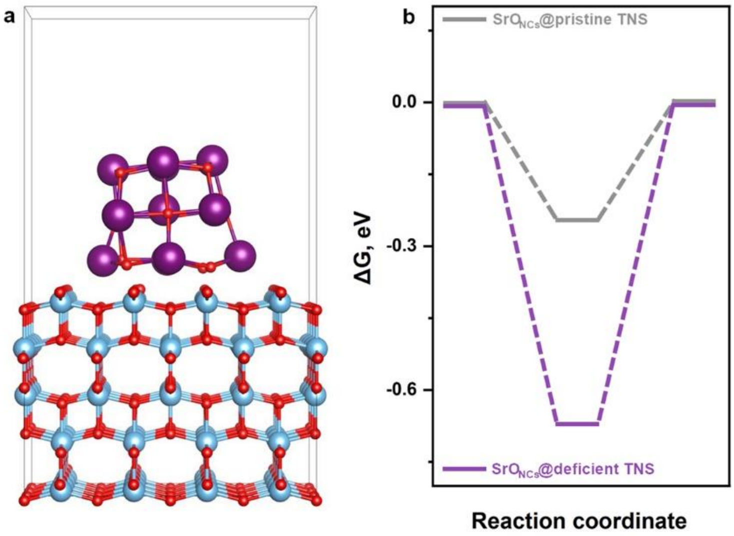

Supplementary Figure 15. a. Relaxed micro-structure of SrO_{NCs} deposited at TNS surface. The blue, red and purple spheres depict Ti, O and Sr atoms respectively. **b.** Calculated binding energy of SrO_{NCs} deposited at pristine and deficient TNS surfaces respectively.

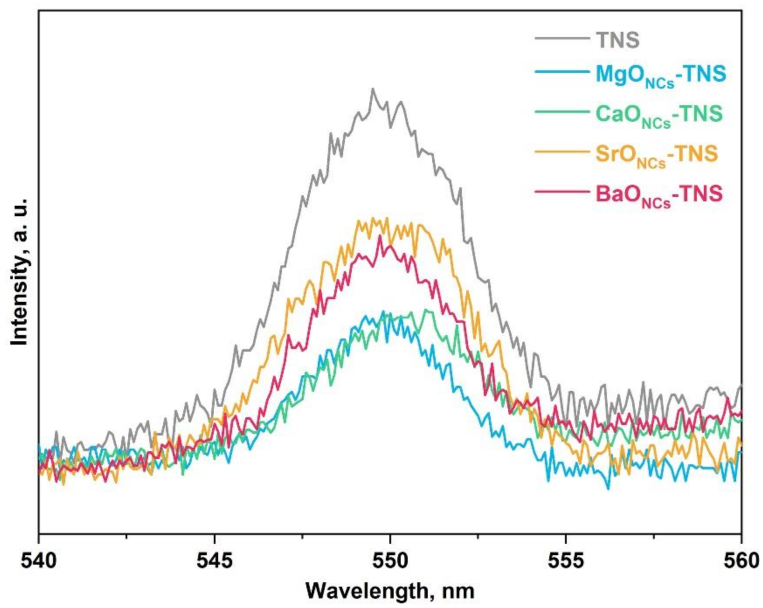

Supplementary Figure 16. PL spectra for pristine TNS, MgO$_{NCs}$-TNS, CaO$_{NCs}$-TNS, SrO$_{NCs}$-TNS and BaO$_{NCs}$-TNS, respectively.

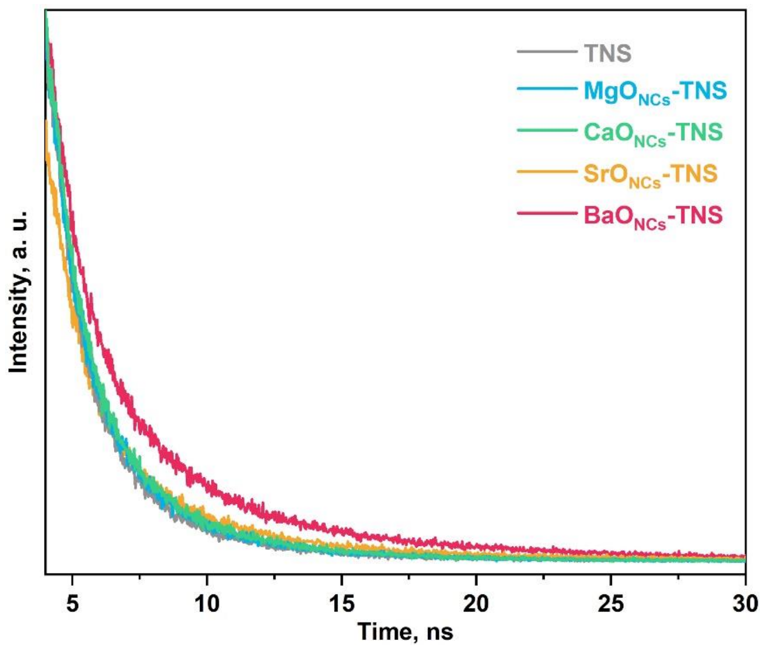

Supplementary Figure 17. Time-resolved fluorescence emission decay spectra for pristine TNS, MgO$_{NCs}$-TNS, CaO$_{NCs}$-TNS, SrO$_{NCs}$-TNS and BaO$_{NCs}$-TNS, respectively.

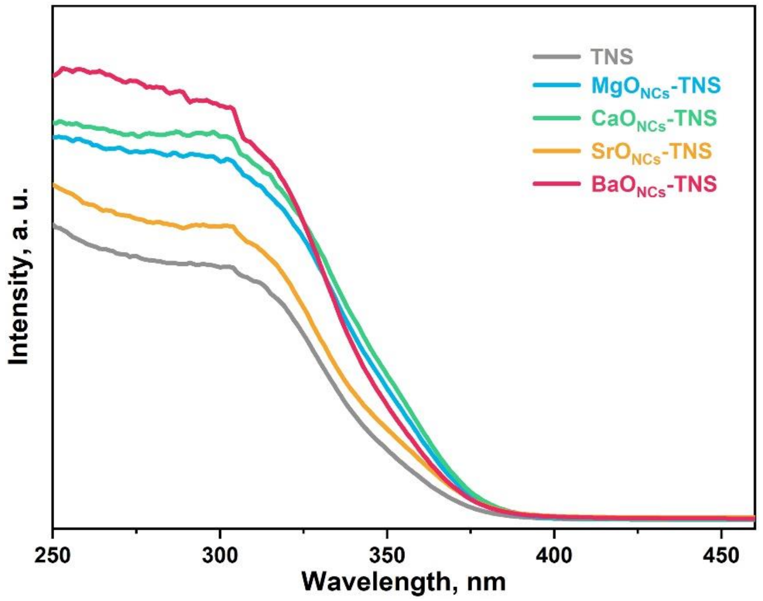

Supplementary Figure 18. UV-vis DRS results for pristine TNS, MgO_{NCs}-TNS, CaO_{NCs}-TNS, SrO_{NCs}-TNS and BaO_{NCs}-TNS, respectively.

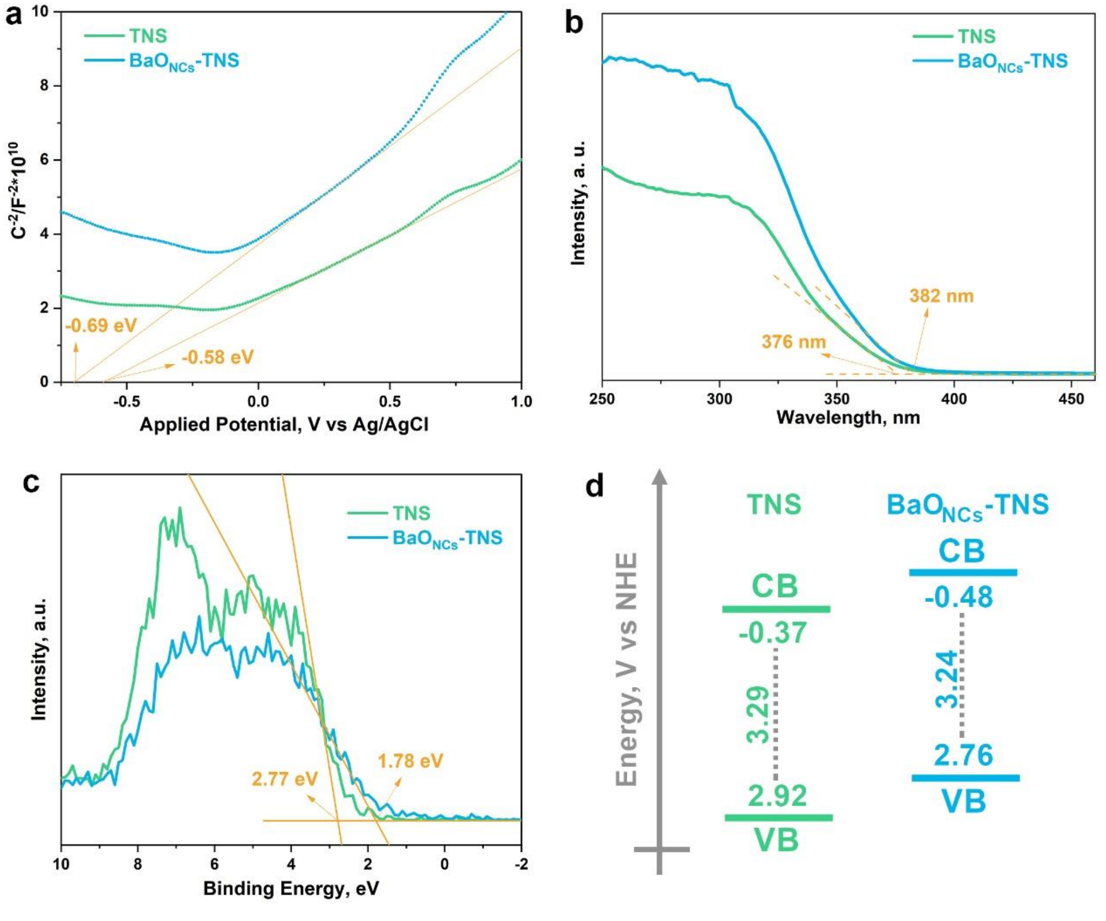

Supplementary Figure 19. Estimated band structures. Mott-Schottky spectra (a), estimated band gap calculated by UV-vis DRS (b), XPS VB spectra (c) and illustration

for the band structures of TNS and BaO_{NCs}-TNS.

Since the contaminative C element is inevitable during the XPS tests, the band edge cannot be precisely characterized by the XPS VB spectra. Hence the Mott-Schottky spectra and UV-vis DRS were combined to determine the band structures of TNS and BaO_{NCs}-TNS.

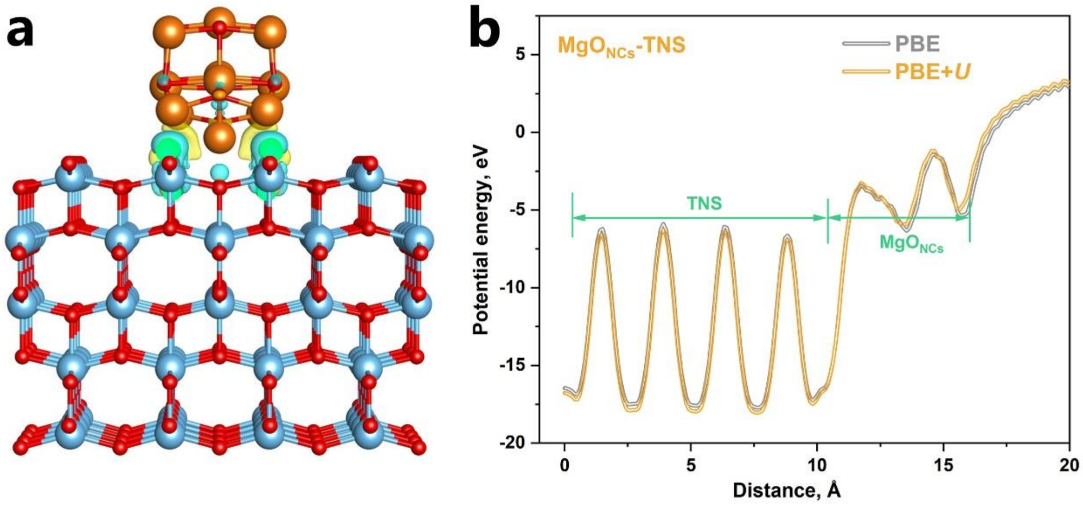

Supplementary Figure 20. Calculated charge difference density **(a)** and planer average potential energy profile **(b)** for the interface between MgO_{NCs} and TNS. The charge accumulation is labeled in blue and charge depletion is labeled in yellow. The isosurface was set to 0.0047 eV Å^{-3}. Blue, red and orange spheres depict Ti, O and Mg atoms, respectively.

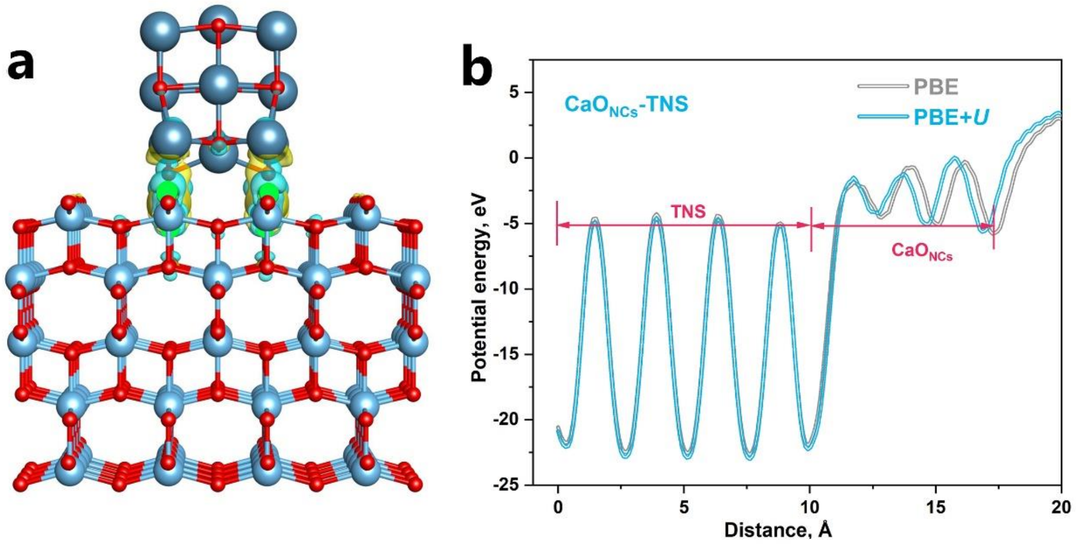

Supplementary Figure 21. Calculated charge difference density **(a)** and planer average potential energy profile **(b)** for the interface between CaO$_{NCs}$ and TNS. The charge accumulation is labeled in blue and charge depletion is labeled in yellow. The isosurface was set to 0.005 eV Å^{-3}. Blue, red and cyan spheres depict Ti, O and Ca atoms, respectively.

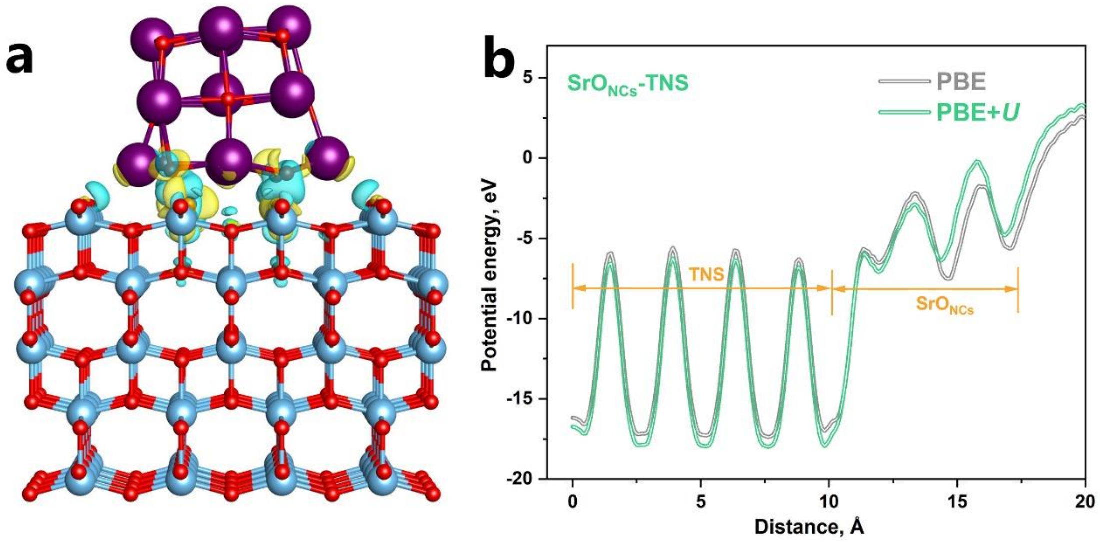

Supplementary Figure 22. Calculated charge difference density **(a)** and planer average potential energy profile **(b)** for the interface between SrO$_{NCs}$ and TNS. The charge accumulation is labeled in blue and charge depletion is labeled in yellow. The isosurface was set to 0.005 eV Å^{-3}. Blue, red and purple spheres depict Ti, O and Sr atoms respectively.

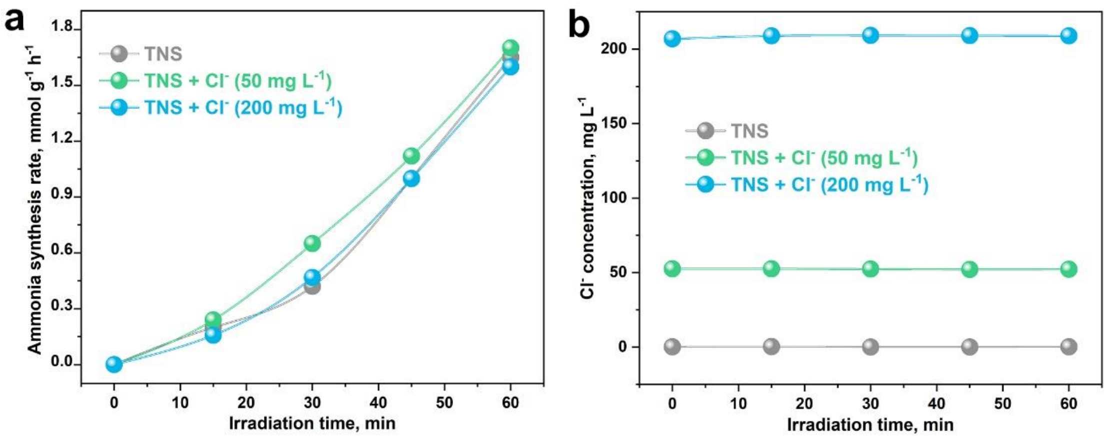

Supplementary Figure 23. Controlled experiment by adding Cl^- into the catalysis system of pristine without other cations or anions.

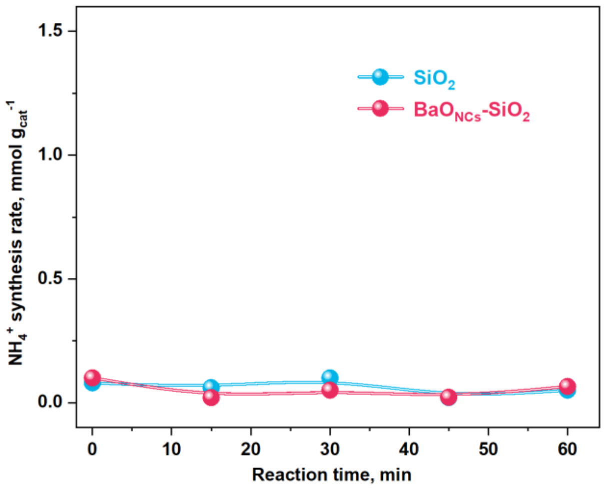

Supplementary Figure 24. Control experiment towards NO_3^-RR to NH_4^+ synthesis by replacing TiO_2 with SiO_2. The test parameters were the same with that of Fig. 3a.

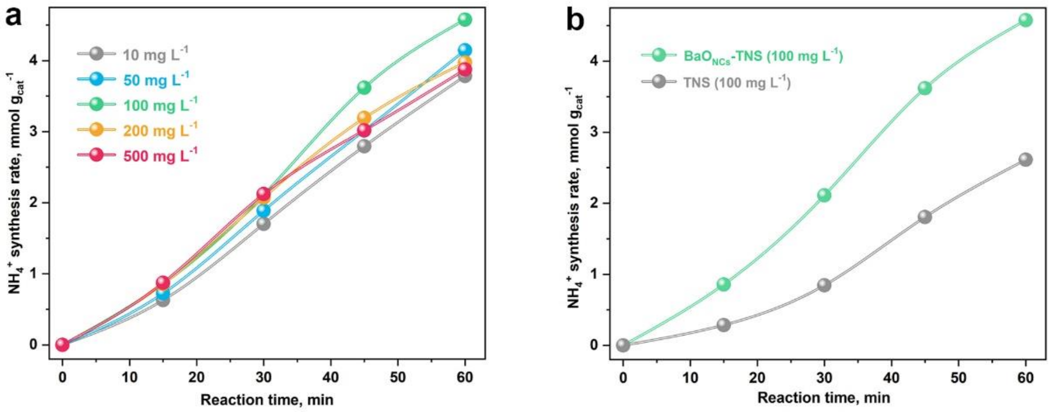

Supplementary Figure 25. Initial NO_3^- concentration optimization on BaO_{NCs}-TNS for the promotion of NH_4^+ photosynthesis efficiency **(a)** and its comparison towards the pristine TNS **(b)**.

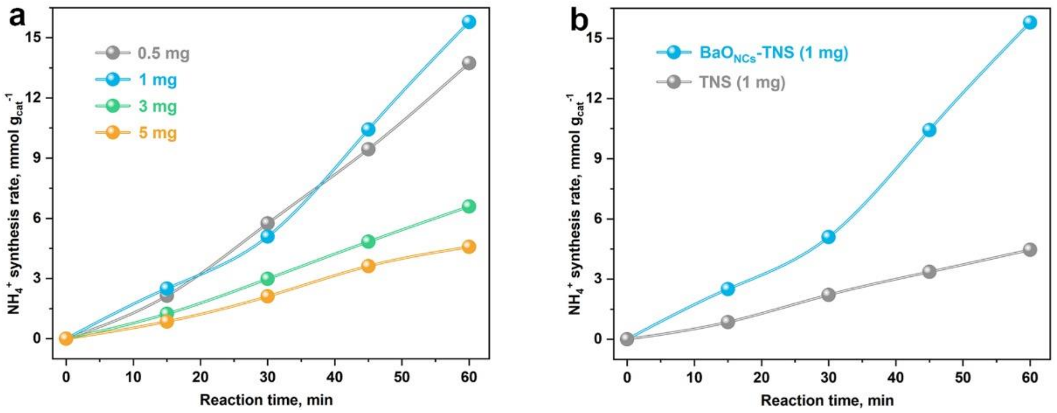

Supplementary Figure 26. Catalyst dosage optimization on BaO_{NCs}-TNS for the promotion of NH_4^+ photosynthesis efficiency **(a)** and its comparison towards the pristine TNS **(b)**.

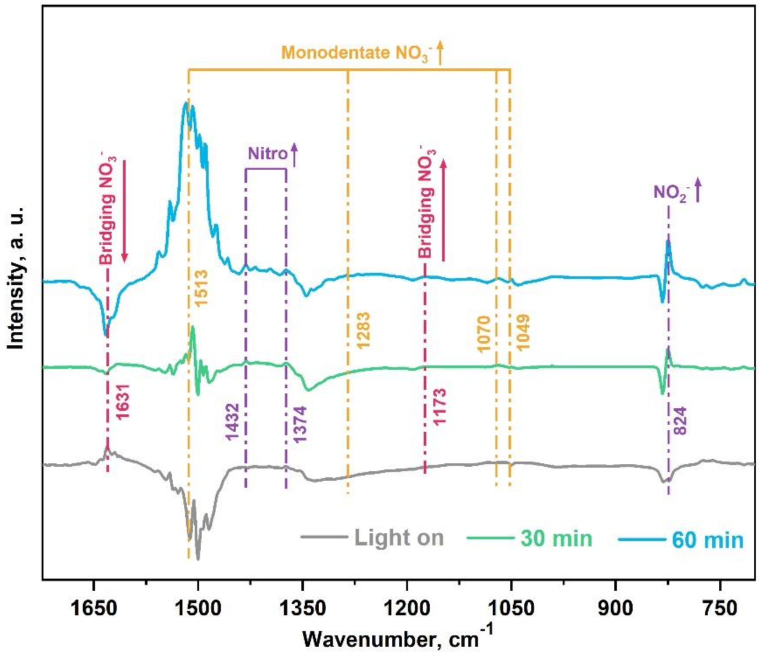

Supplementary Figure 27. *In-situ* DRIFTS results for the preactivation of NO_3^- by UV light irradiation.

The test procedure is similar with that of the in-situ DRIFTS test in Fig. 5b by replacing the NO_3^--contained catalyst with pristine KNO_3 powder. It is observed in the above figure that some bridging NO_3^- (1631 and 1173 cm^{-1})[1, 2] is transferred into the monodentate NO_3^- species (1513, 1283, 1070 and 1049 cm^{-1})[2, 3], which is more active than that of the bridging NO_3^- [4]. Moreover, the intermediated nitro (-NO_2, 1432 and 1374 cm^{-1})[4] and NO_2^- (824 cm^{-1}) are obviously increased. It is concluded that the NO_3^- reactant can be preactivated by UV light irradiation.

Supplementary reference:

1 Zhang, X. L., He, H, Cao, H. W. & Yu, Y. B. Experimental and theoretical studies of surface nitrate species on Ag/Al_2O_3 using DRIFTS and DFT. *Spectrochim. Acta A*, **71**, 1446-1451 (2008).

2 Yang, X. F. *et al.* DRIFTS Study of Ammonia Activation over CaO and Sulfated CaO for NO Reduction by NH_3. *Environ. Sci. Technol.*, **45**, 1147-1151 (2011).

3 Cui, W. *et al*. Highly Efficient Performance and Conversion Pathway of Photocatalytic NO Oxidation on SrO-Clusters@Amorphous Carbon Nitride. *Environ. Sci. Technol.*, **51**, 10682-10690 (2017).

4 Hadjiivanov, K. Identification of Neutral and Charged N_xO_y Surface Species by IR

Spectroscopy. *Catal. Rev. Sci. Eng.*, **42**, 71-144 (2000).

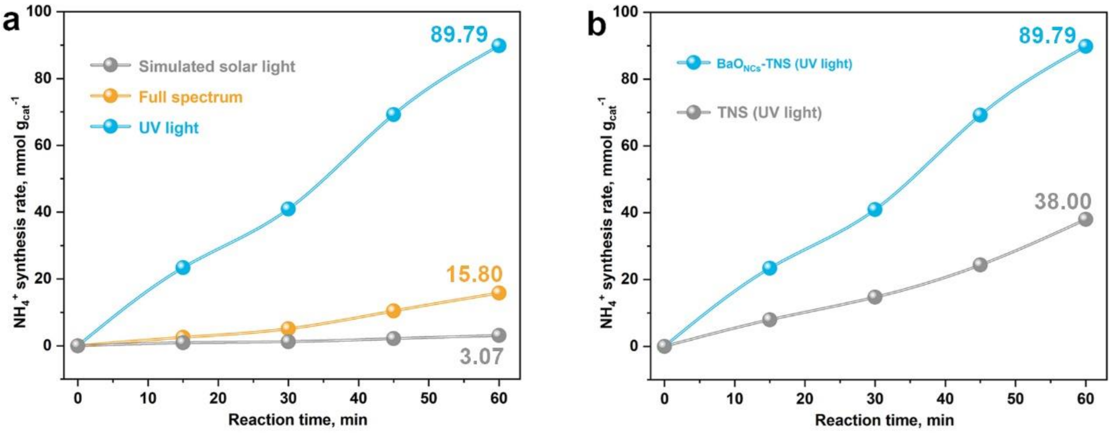

Supplementary Figure 28. Light source optimization on BaO$_{NCs}$-TNS for the promotion of NH_4^+ photosynthesis efficiency **(a)** and its comparison towards the pristine TNS **(b)**.

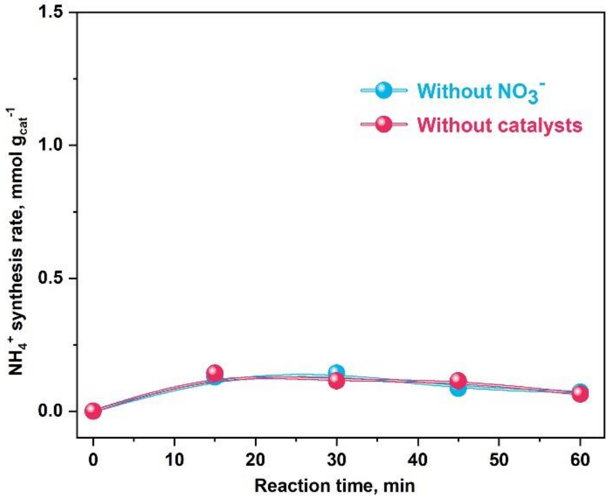

Supplementary Figure 29. Blank control experiment excluding the contribution of contaminative N source to NH_4^+ photosynthesis by conducting the photocatalytic activity evaluation without NO_3^- and catalysts respectively.

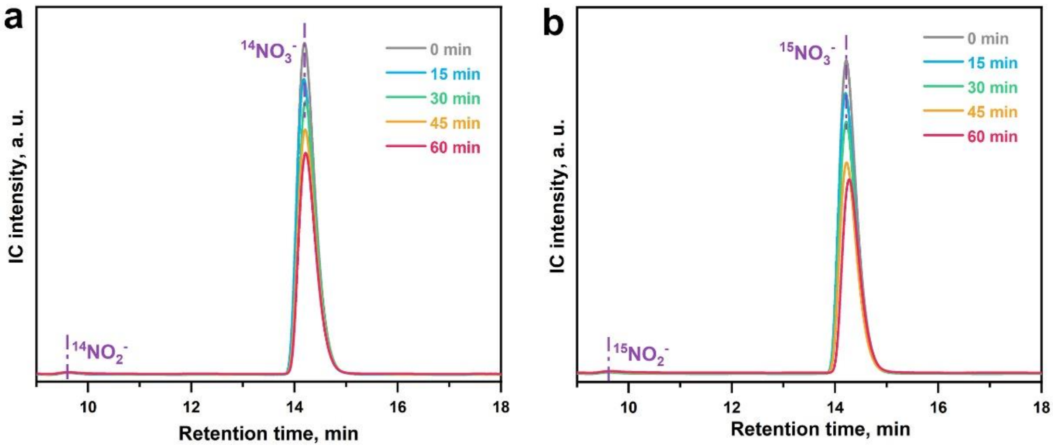

Supplementary Figure 30. Detected IC signals for $^{14}NO_3^-$/$^{14}NO_2^-$ **(a)** and $^{15}NO_3^-$/$^{15}NO_2^-$ **(b)** during the reaction process. The ranges of y axis were set consistently for both **(a)** and **(b)**.

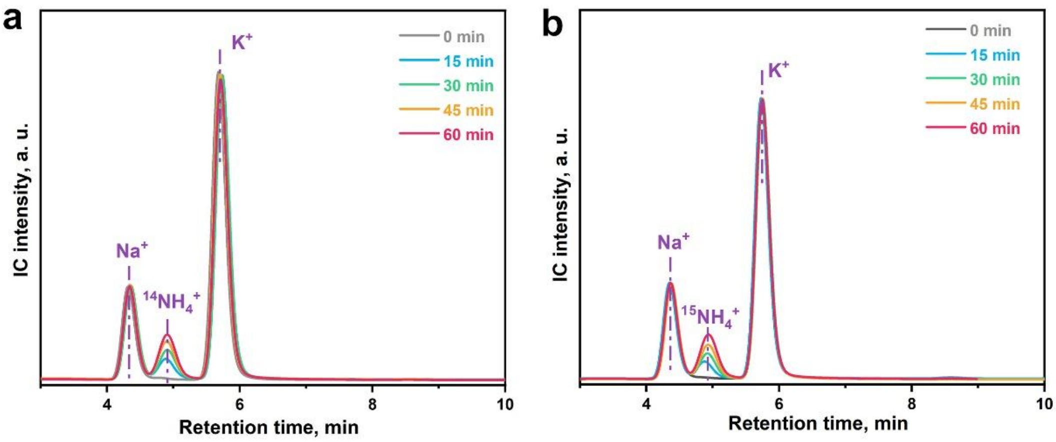

Supplementary Figure 31. Detected IC signals for $^{14}NH_4^+$ **(a)** and $^{15}NH_4^+$ **(b)** during the reaction process. The ranges of y axis were set consistently for both **(a)** and **(b)**.

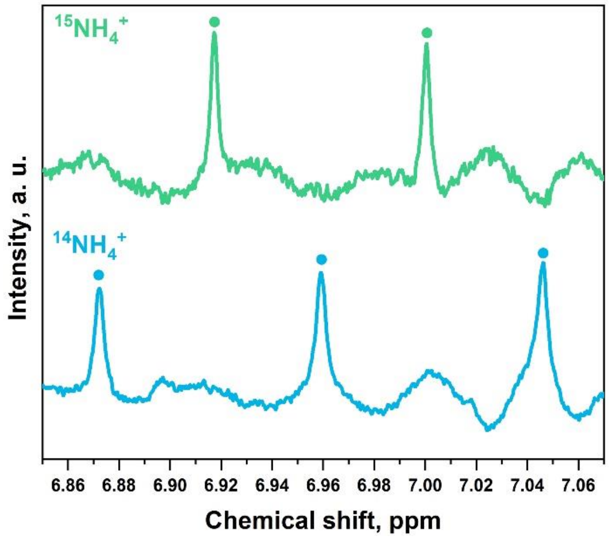

Supplementary Figure 32. ^1H NMR spectra of the produced NH_4^+ using $^{14}NO_3^-$ and $^{15}NO_3^-$ as the nitrogen source respectively.

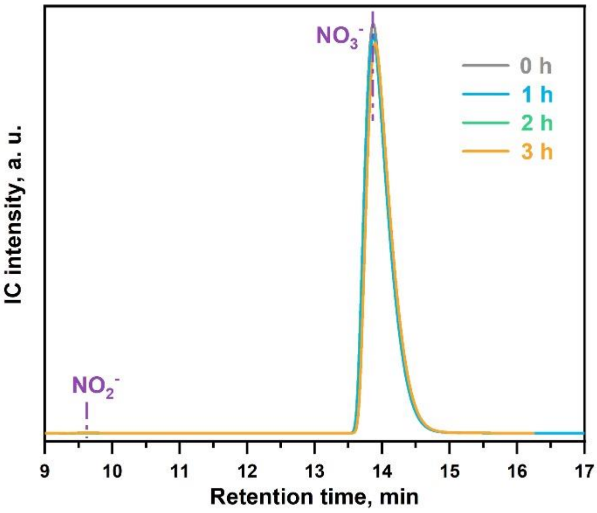

Supplementary Figure 33. Detected IC signals for NO_3^- consumption and NO_2^- generation in the selectivity tests.

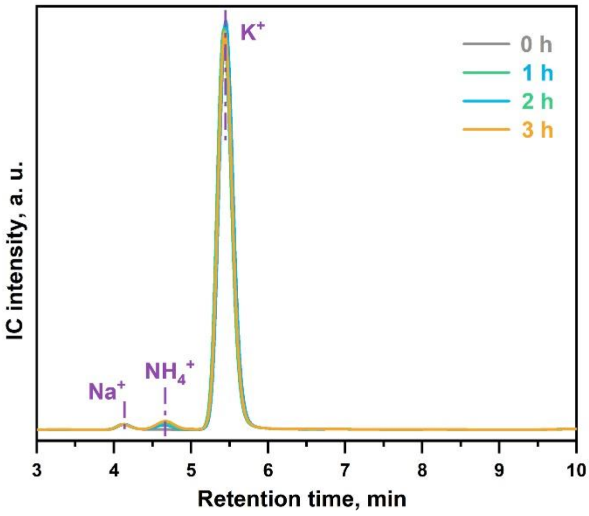

Supplementary Figure 34. Detected IC signals for NH_4^+ generation in the selectivity tests.

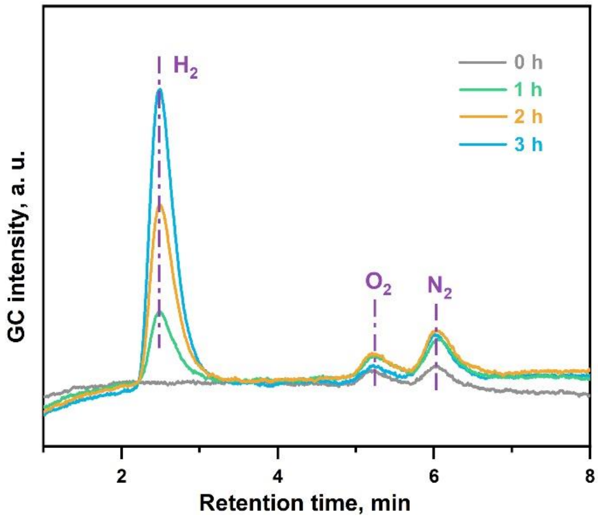

Supplementary Figure 35. Detected GC signals for H_2 generation in the selectivity tests.

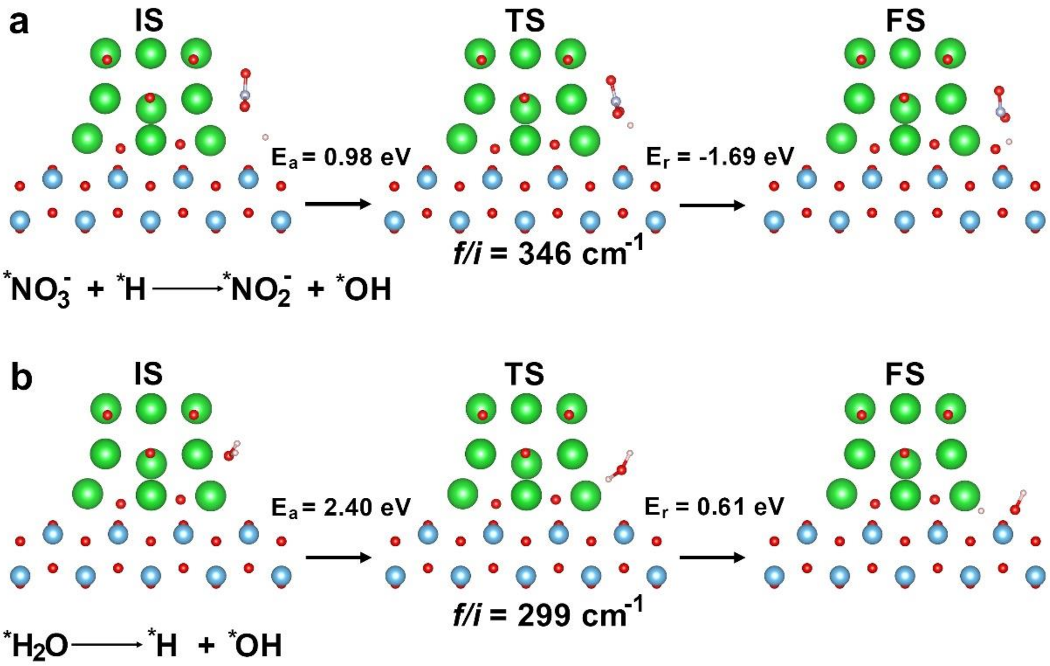

Supplementary Figure 36. Calculated reaction coordinates of NO_3^- reduction **(a)** and water splitting **(b)**. The transition state was identified when a single imaginary frequency was located and all forces on atoms were zero. The blue, red, green, light grey and pink spheres depict Ti, O, Ba, N and H atoms, respectively.

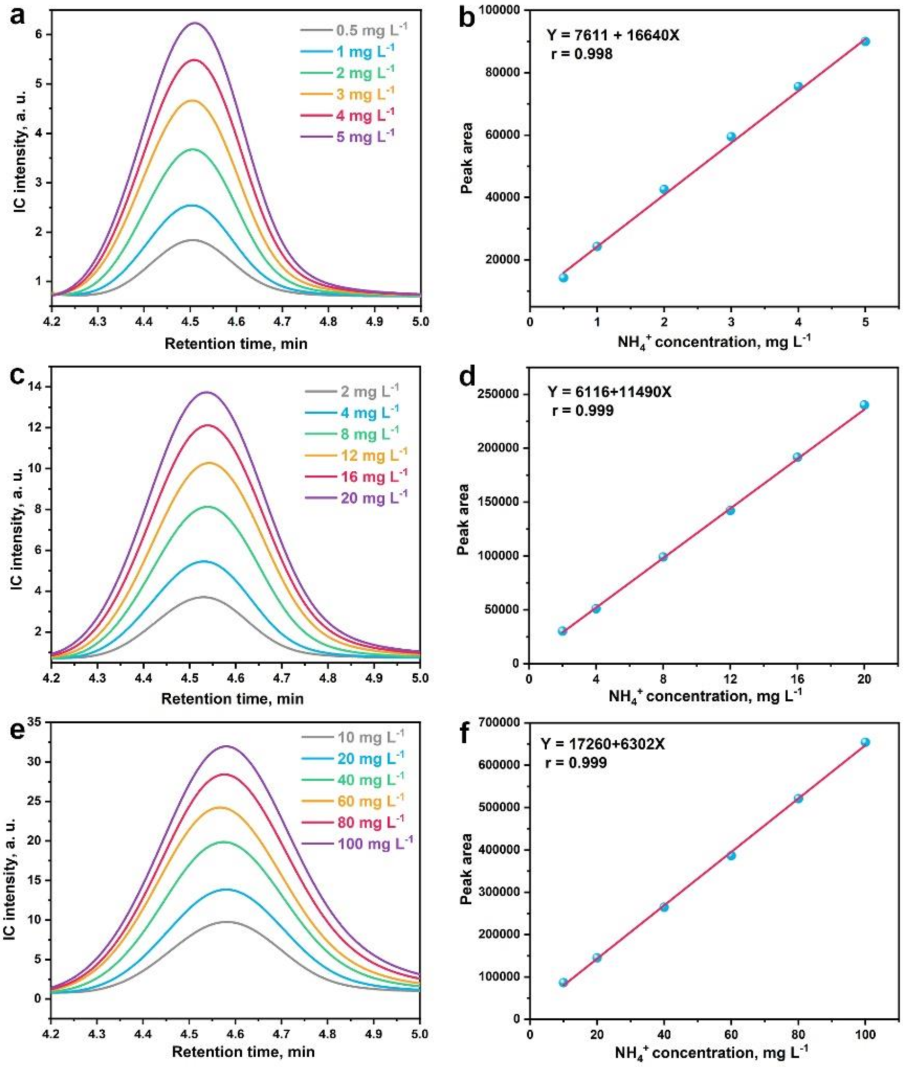

Supplementary Figure 37. Plotting of standard curves of NH_4^+ in the concentration range of 0.5-5 mg L^{-1} (**a and b**), 2-20 mg L^{-1} (**c and d**) and 10-100 mg L^{-1} (**e and f**) respectively by IC detection.

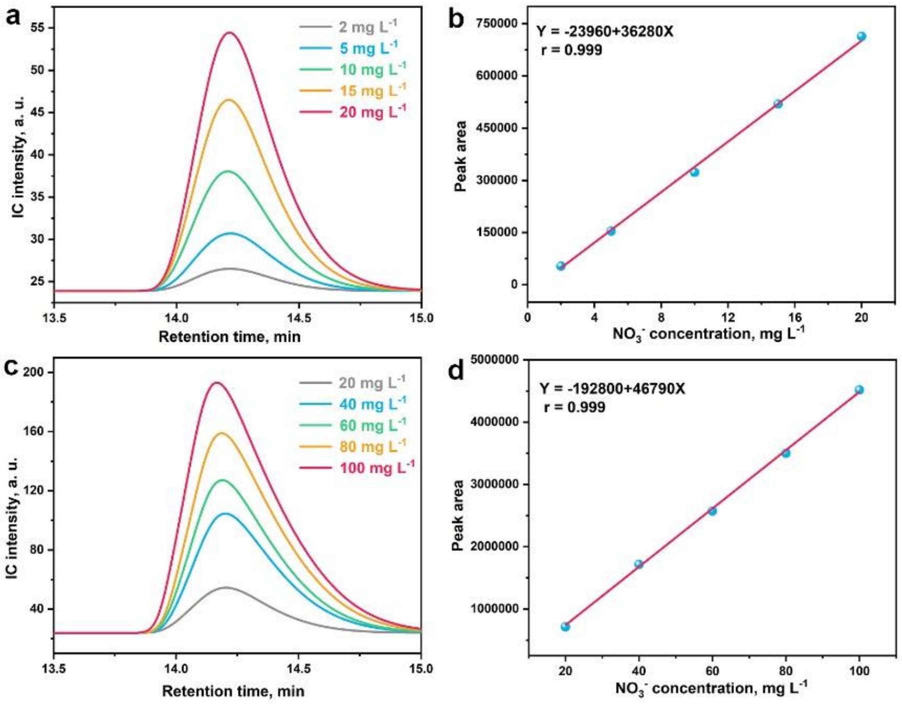

Supplementary Figure 38. Plotting of standard curves of NO_3^- in the concentration range of 2-20 mg L^{-1} (**a and b**) and 20-100 mg L^{-1} (**c and d**) respectively by IC detection.

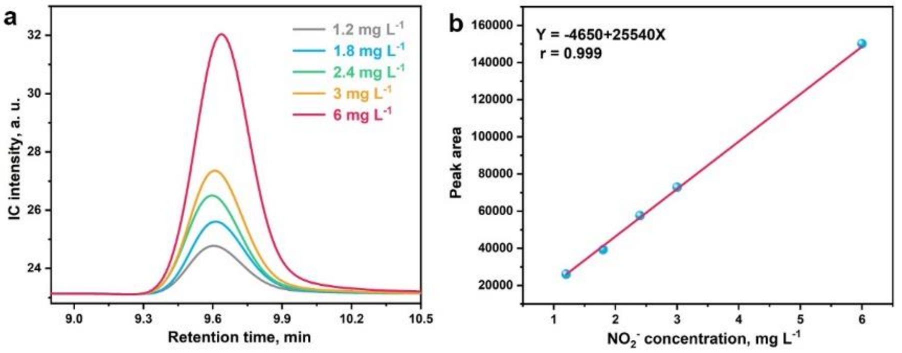

Supplementary Figure 39. Plotting of standard curves of NO_2^- in the concentration range of 1.2-6 mg L^{-1} by IC detection.

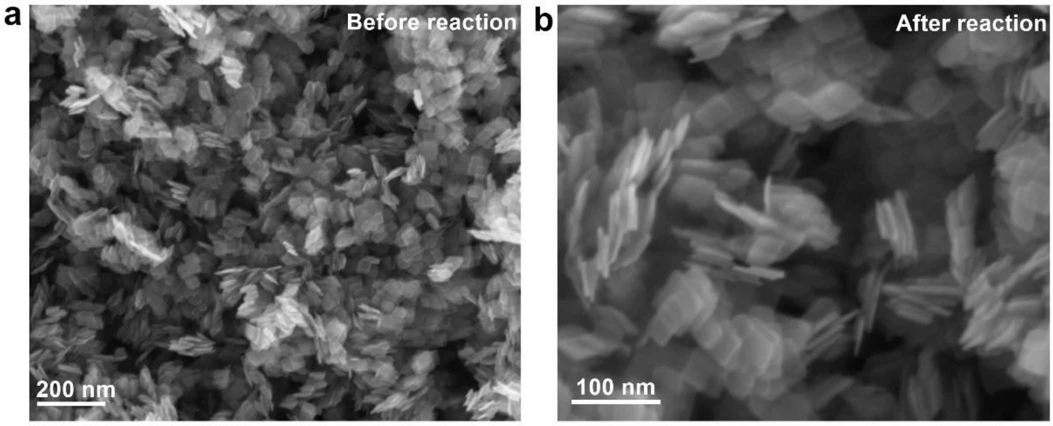

Supplementary Figure 40. SEM images for BaO_{NCs}-TNS before **(a)** and after **(b)** the NH_4^+ photosynthesis reaction.

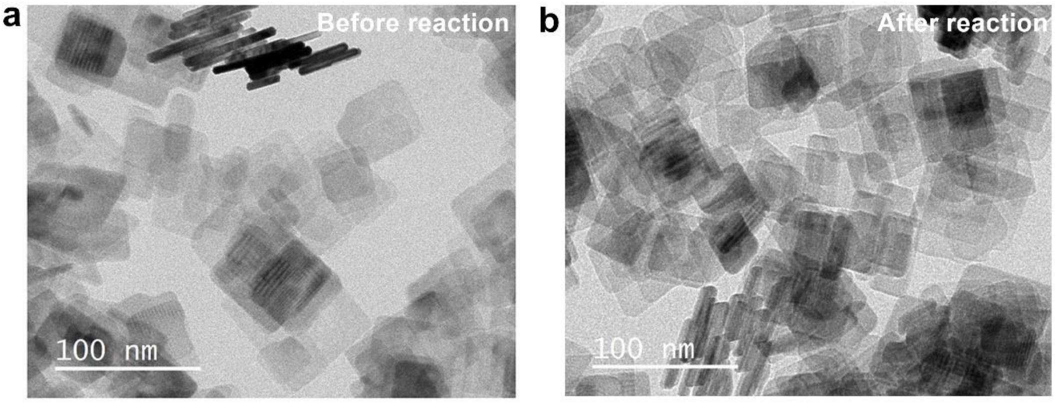

Supplementary Figure 41. TEM images for BaO_{NCs}-TNS before **(a)** and after **(b)** the NH_4^+ photosynthesis reaction.

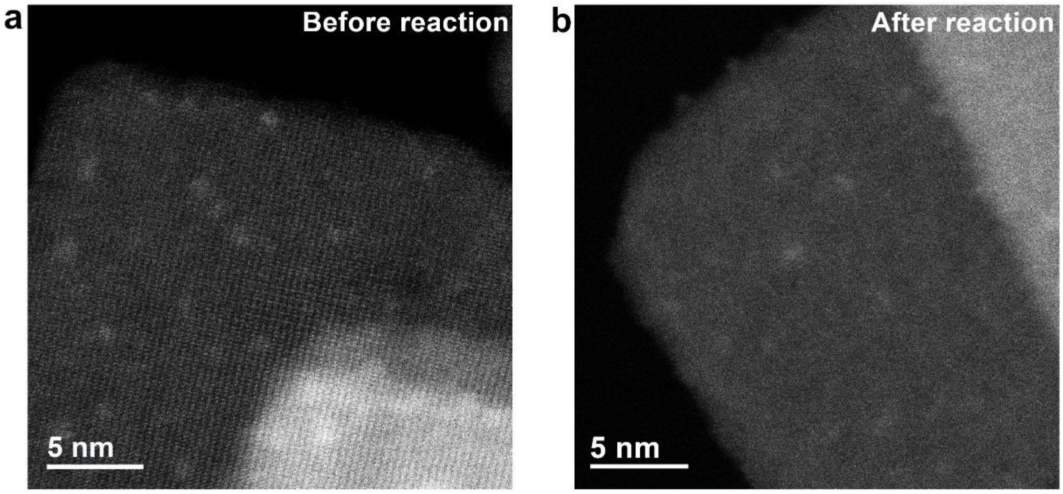

Supplementary Figure 42. HAADF-STEM images for BaO_{NCs}-TNS before **(a)** and after **(b)** the NH_4^+ photosynthesis reaction.

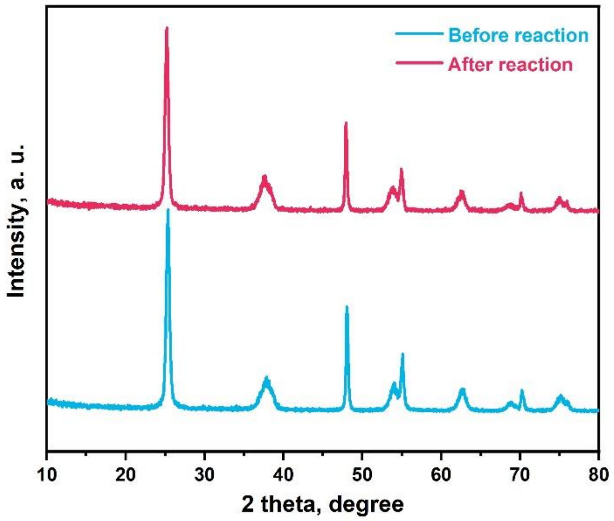

Supplementary Figure 43. XRD patterns for BaO_{NCs}-TNS before and after the NH_4^+ photosynthesis reaction.

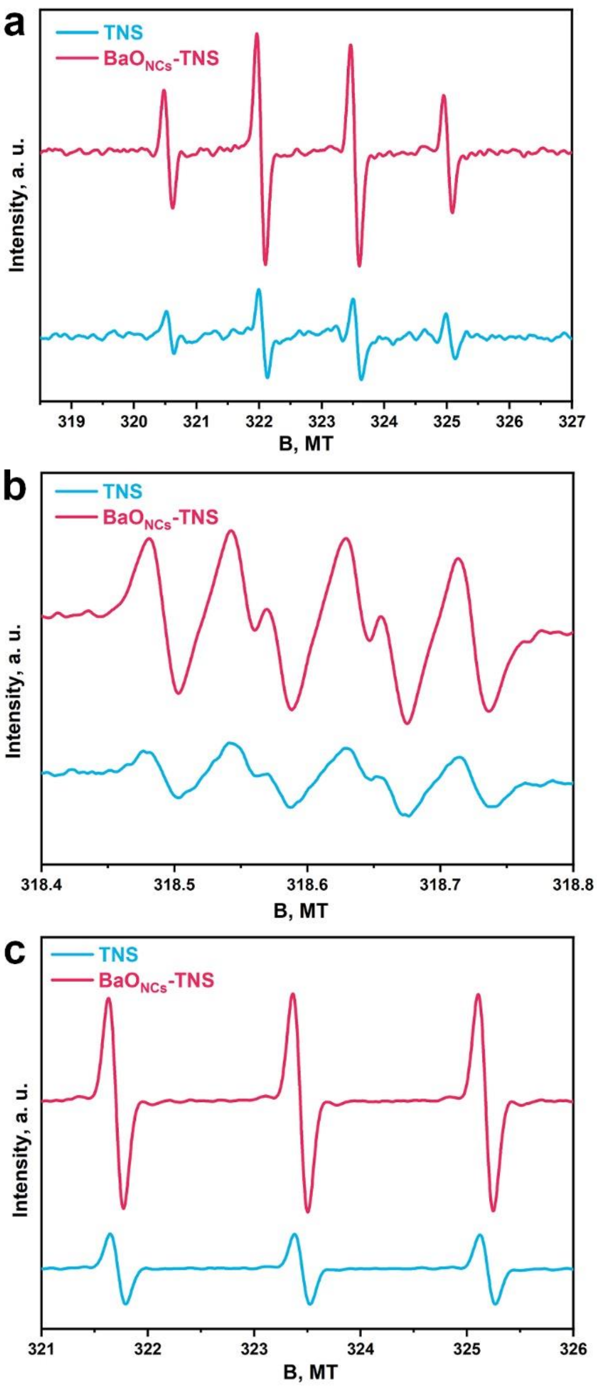

Supplementary Figure 44. Liquid-state EPR results for the detection of light-generated $^{\bullet}OH$ (**a**), $^{\bullet}O_2^-$ (**b**) and $^1O_2$ (**c**) radicals on the pristine and BaO_{NCs} deposited TNS.

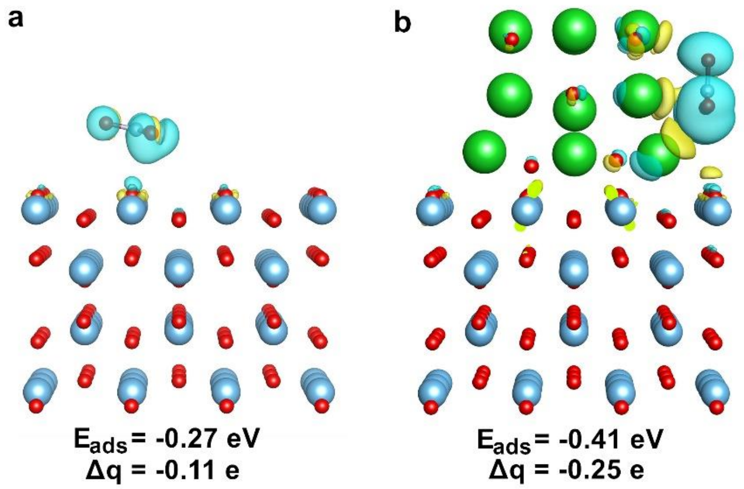

Supplementary Figure 45. Calculated results for NO_3^- adsorption at the pristine (a) and BaO_{NCs} deposited (b) TNS respectively. The blue, red, green and light grey spheres depict Ti, O, Ba and N atoms, respectively. The charge accumulation is labeled in blue and charge depletion is labeled in yellow. The isosurface was set to 0.015 eV Å^{-3}.

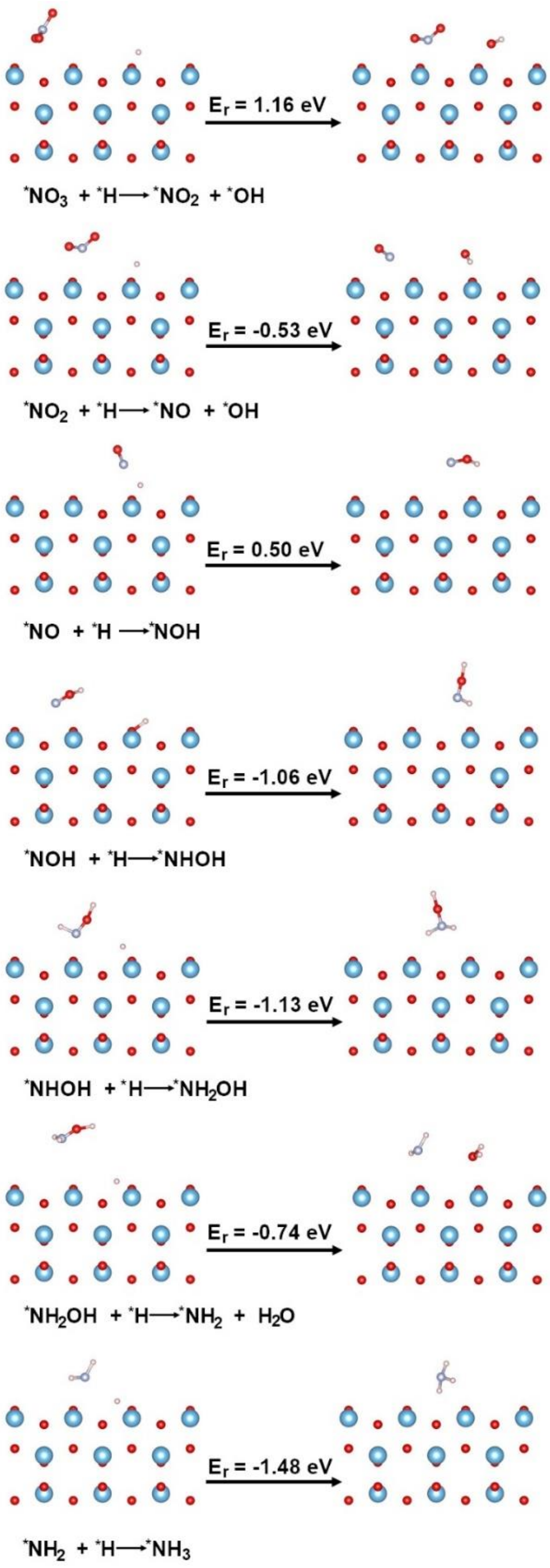

Supplementary Figure 46. Calculated reaction coordinates for NO_3^- reduction at the pristine TNS surface (corresponding to the green line in Fig. 5c). The blue, red, light grey and pink spheres depict Ti, O, N and H atoms, respectively.

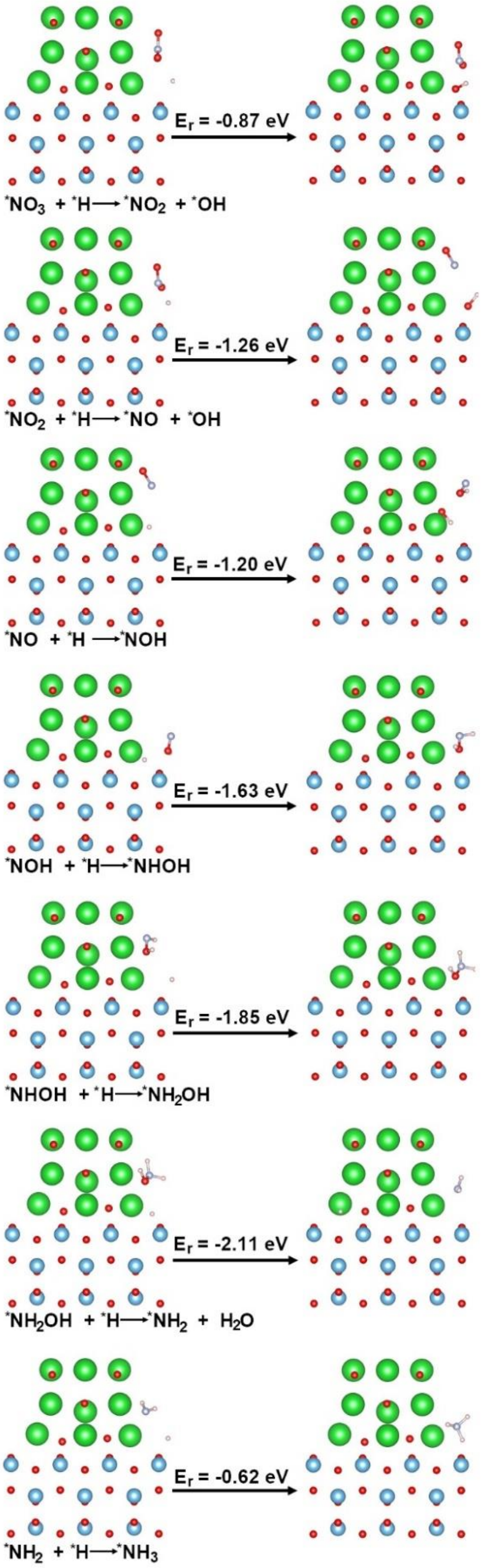

Supplementary Figure 47. Calculated reaction coordinates for NO_3^- reduction at the BaO_{NCs}-TNS surface (corresponding to the blue line in Fig. 5c). The blue, red, green, light grey and pink spheres depict Ti, O, Ba, N and H atoms, respectively.

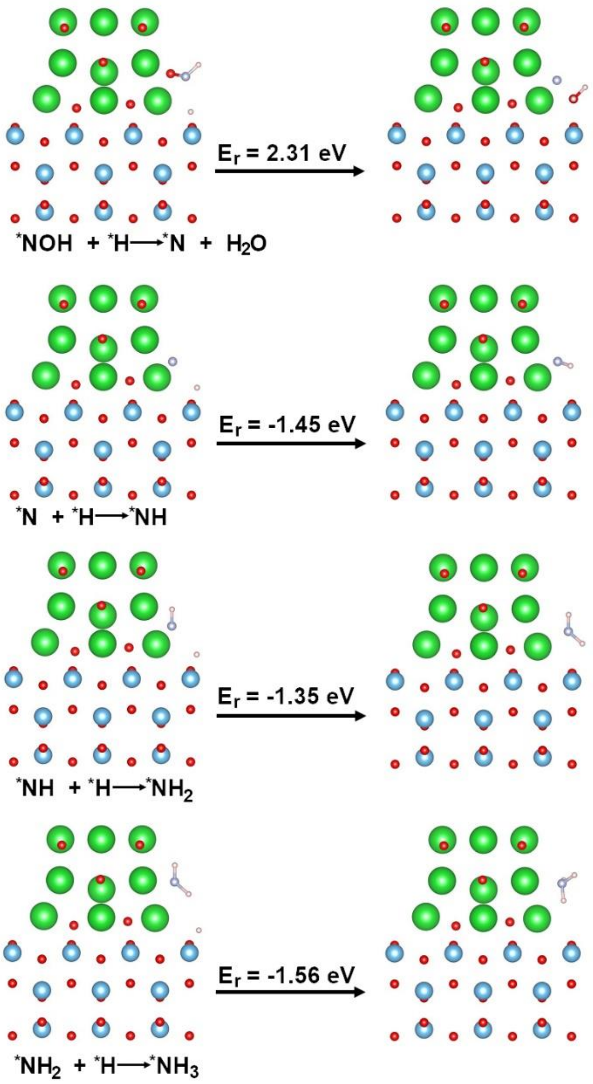

Supplementary Figure 48. Calculated reaction coordinates for the potential side reactions at the BaO_{NCs}-TNS surface (corresponding to the red line in Fig. 5c). The blue, red, green, light grey and pink spheres depict Ti, O, Ba, N and H, atoms respectively.

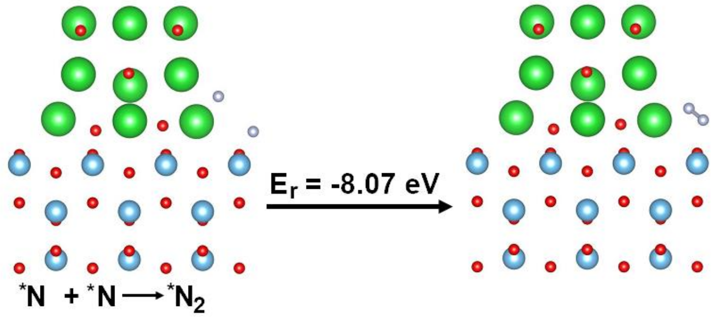

Supplementary Figure 49. Calculated reaction coordinates for the potential side reactions at the BaO_{NCs}-TNS surface (corresponding to the purple line in Fig. 5c). The blue, red, green, light grey and pink spheres depict Ti, O, Ba, N and H atoms, respectively.

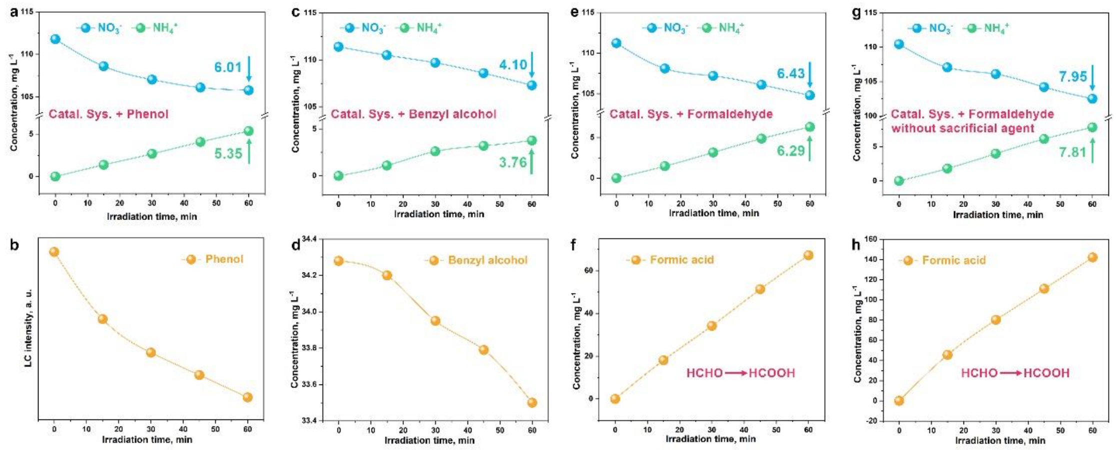

Supplementary Figure 50. Raw data of the efficiency evaluation in simulated wastewater containing the phenol (a, b), benzyl alcohol (c, d) and formaldehyde (e, f) as organic contaminants. The EG is excluded as hole sacrificial agent for the formaldehyde test (g ,h)

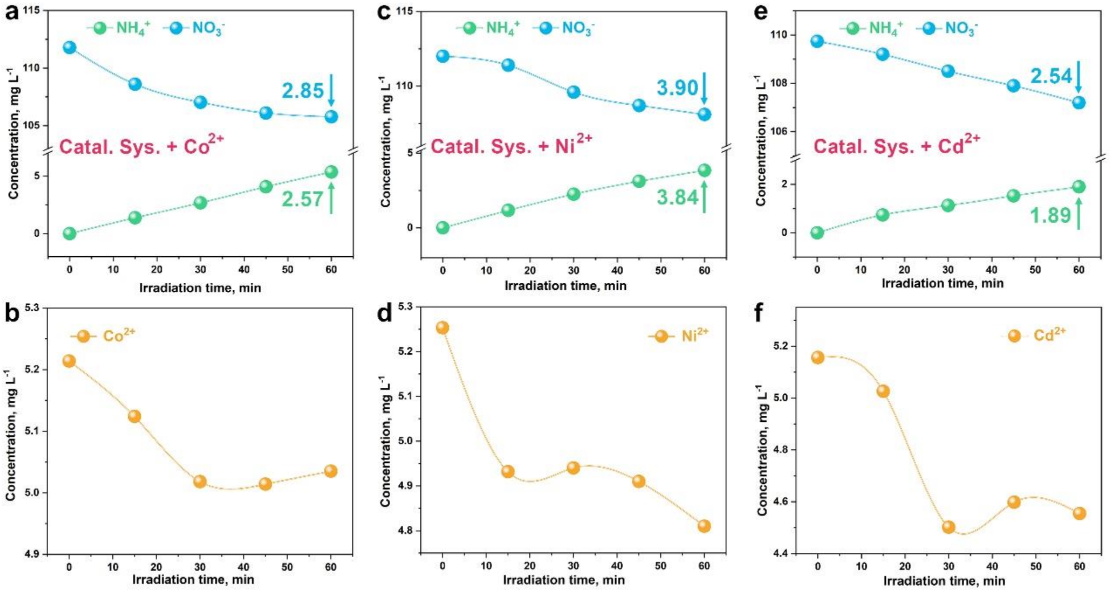

Supplementary Figure 51. Raw data of the efficiency evaluation in simulated wastewater containing the Co^{2+} (a, b), Ni^{2+} (c, d) and Cd^{2+} (e, f) as cation contaminants.

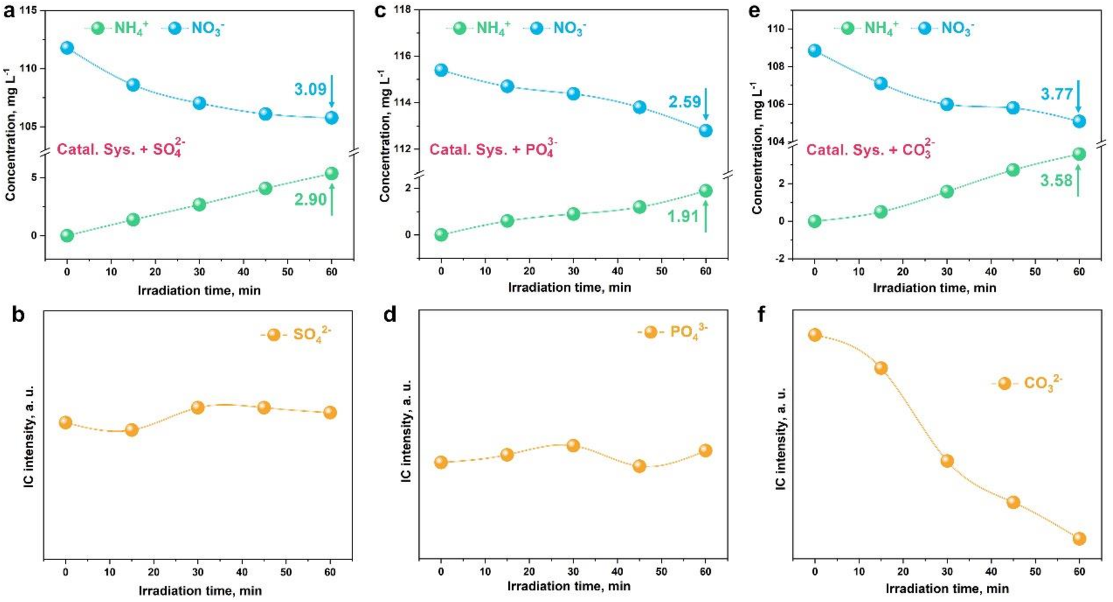

Supplementary Figure 52. Raw data of the efficiency evaluation in simulated wastewater containing the SO_4^{2-} (a, b), PO_4^{3-} (c, d) and CO_3^{2-} (e, f) as anion contaminants.

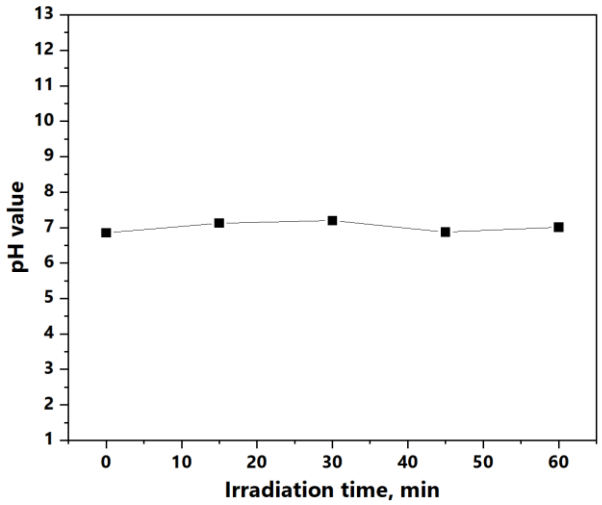

Supplementary Figure 53. pH value variation during the catalytic test.

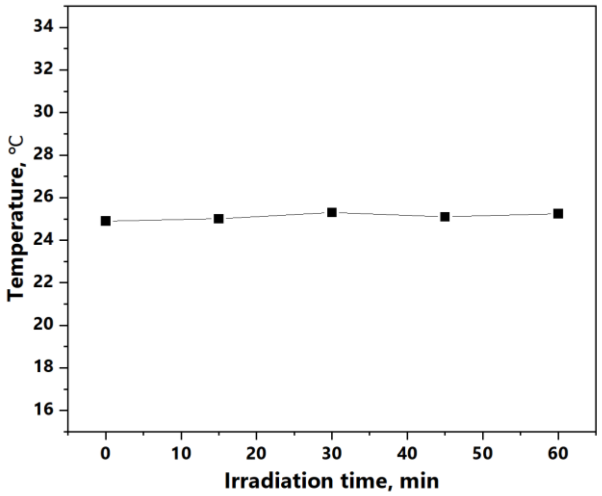

Supplementary Figure 54. Temperature variation during the catalytic test.

Supplementary Note 1

Apparent quantum yield calculation details

●Photo flux calculation

The photon flux in our photocatalysis measurements was obtained based on the following formulas:

$$\Theta = \frac{N}{ST}$$

$$\mathrm{N} = \frac{PT\ \bar{\lambda}}{hc}$$

$$\mathrm{P} = \bar{\mathrm{E}}\mathrm{S}$$

$$\bar{\lambda} = \frac{\int_{\Delta\lambda} \lambda E(\lambda) d\lambda}{E}$$

where Θ is the photo flux, *N* is the incident photon number, *S* stands for the illumination area of 3.0 cm^{-2}, *T* refers to the illumination time, *h* corresponds to the Planck constant, c stands for the speed of light, λ refers to wavelength, $\bar{\lambda}$ refers to an average wavelength (in this work the equivalent $\bar{\lambda}$ is 524.5 nm according to the MC-X301B light source used in this work, provided by the Merry Change Technology Co., Ltd.), $\bar{E}$ is the average optical power density measured by the optical power meter (74.6 mW cm^{-2}), E is total radiation intensity, E(λ) is the spectrum radiation intensity. Thus, the photon flux Θ could be $1.97 \times 10^{17}\ s^{-1}\ cm^{-2}$.

●Apparent quantum efficiency calculation

The quantum efficiency is defined by the ratio of the effective electrons used for product formation to the total input photon flux. The reduction of NO_3^- to NH_4^+ molecule requires eight electrons:

$$\mathrm{QE\%} = \frac{Effective\ electrons}{Total\ photons} \times 100\% = 8\mathrm{X}N/\Theta TS \times 100\%$$

where *X* is the yields of NH_4^+, respectively. *N* is Avogadro's number, *Θ* is the photon flux, *T* is the irradiation time, and *S* is the illumination area. The following calculation example is based on the data from NO_3^-RR with BaO_{NCs}-TNS for 1h (Fig. 3a): $X=8.25\times10^{-3}$ mol, $N=6.022\times10^{23}$ mol^{-1}, T=1 h, $S=3.0$ cm^2; equivalent $\Theta=1.97\times10^{17}$ s^{-1} cm^{-2}.

QE% = (8 × 8.25×10^{-3} mol × 6.022×10^{23} mol^{-1})/(1.97×10^{17} s^{-1} cm^{-2} × 1 × 3600 s × 3.0 cm^{2}) = 0.53%

Supplementary Note 2

Competing reaction pathways of NO_3^- reduction for NH_4^+ photosynthesis (corresponding to the red line in Fig. 5c).

$$*NOH + {}^{\bullet}H \rightarrow *N + *H_2O \quad (1)$$

$$*N + {}^{\bullet}H \rightarrow *NH \quad (2)$$

$$*NH + {}^{\bullet}H \rightarrow *NH_2 \quad (3)$$

$$*NH_2 + {}^{\bullet}H \rightarrow *NH_3 \quad (4)$$

Supplementary Note 3

Potential side reaction pathways of NO_3^- reduction into N_2 (corresponding to the purple and blue line in Fig. 5c).

$$*N + *N \rightarrow *N_2 \quad (5)$$

Supplementary Table 1 Comparison of the ammonia synthesis efficiency between NO_3^-RR to ammonia photosynthesis catalyzed by BaO_{NCs} in this work and recently reported alkaline earth-based catalysts for ammonia synthesis.

Ammonia synthesis route	Catalyst	Optimal ammonia synthesis rate (mmol g_{cat}^{-1} h^{-1})	Reference
**NO_3^-RR to ammonia Photosynthesis**	**BaO_{NCs}-TNS**	**89.79**	**This work**
Photocatalytic N_2RR to ammonia	BMOF(Sr)−0.2Fe	0.78	*ACS Catal.*, **2021**, *11*, 9986-9995.
Photocatalytic N_2RR to ammonia	g-C_3N_4/ $Mg_{1.1}Al_{0.3}Fe_{0.2}O_{1.7}$	0.27	*RSC Adv.*, **2021**, *7*, 18099-18107.
Haber–Bosch process T = 360 °C P = 0.9 MPa	Ru/Ba-Ca$(NH_2)_2$	60.4	*Angew. Chem. Int. Ed.*, **2018**, *57*, 2648-2652.
Haber–Bosch process T = 400 °C P = 10.0 MPa	Ba–Ru/CNTs-D	49.0	*J. Energy Chem.*, **2020**, *41*, 79-86.
Haber–Bosch process T = 340 °C P = 1.0 MPa	Ru/BaO-CaH_2	10.5	*ACS Catal.*, **2018**, *8*, 10977-10984.
Haber–Bosch process T = 400 °C P = 0.1 MPa	Ru/$Sr_2Nb_2O_7$	4.98	*Appl. Catal. A: Gen.*, **2018**, *554*, 1-9.
Haber–Bosch process T = 400 °C P = 0.1 MPa	Cs- Ru/$Sr_2Ta_2O_7$	5.04	*J. Catal.*, **2020**, *389*, 556-565.
Haber–Bosch process T = 350 °C P = 0.9 MPa	Ru/Ca$(NH_2)_2$	31.7	*ACS Catal.*, **2016**, *6*, 7577-7584.
Haber–Bosch process T = 340 °C P = 0.1 MPa	Ru-Cs/MgO	1.65	*ACS Catal.*, **2014**, *4*, 674-680.
Haber–Bosch process T = 300 °C P = 1.0 MPa	LiH/Co-Mg-O-co	19	*Chem. Comm.*, **2021**, *574*, 8576-8579.
Haber–Bosch process T = 400 °C P = 1.0 MPa	Ba–Ru/MgO	25.2	*J. Catal.*, **2003**, *214*, 327-335.

Supplementary Table 2 Comparison of the ammonia synthesis efficiency between NO_3^-RR to ammonia photosynthesis in this work and recently reported state-of-the-art ammonia synthesis routes under ambient conditions.

**Ammonia synthesis route**	**Catalyst**	**Optimal ammonia synthesis rate (mmol g_{cat}^{-1} h^{-1})**	**Selectivity or Faraday efficiency (%)**	**long-term stability time (h)**	**Total ammonia synthesis yield (mmol)**	**Reference**
**NO_3^-RR to ammonia Photosynthesis**	**BaO_{NCs}-TNS**	**89.79**	**97.67**	**72**	**0.78**	**This work**
Photocatalytic NO_3^-RR to ammonia	JRC-TIO-6	0.016	97	24	0.384	*ACS Catal.*, **2017**, *7*, 3713-3720.
Photocatalytic NO_3^-RR to ammonia	Ag_2O/P25	0.45	36.9	32	0.28	*Appl. Catal. B: Environ.*, **2015**, *176-177*, 53-61.
Photocatalytic NO_3^-RR to ammonia	Au/TiO_2	0.69	39.0	/	/	*Catal. Today*, **2012**, *181*, 171-176.
Photocatalytic NO_3^-RR to ammonia	PdSn/NiO/$NaTaO_3$	36	72	/	/	*J. Catal.*, **2018**, *361*, 303-312.
Photocatalytic N_2RR to ammonia	OVs-Bi_5O_7Br	12.72	/	0.5	0.16	*J. Am. Chem. Soc.*, **2020**, *142*, 12430-12439.
Photocatalytic N_2RR to ammonia	K^+@g-C_3N_4 With cyano groups	3.42	/	4	0.14	*Angew. Chem. Int. Ed.*, **2019**, *58*, 16644-16650.

Photocatalytic N_2RR to ammonia	Etched ZnCr-LDH nanosheets	0.03	/	3	0.003	*Adv. Energy Mater.*, **2020**, *10*, 2002199.
Photocatalytic N_2RR to ammonia	TiO_2 nanosheets with oxygen vacancies	0.08	/	3	0.005	*Adv. Mater.*, **2019**, *31*, 1806482.
Photocatalytic N_2RR to ammonia	Au@UiO-66	0.81	/	24	0.29	*J. Am. Chem. Soc.*, **2021**, *143*, 5727-5736.
Photocatalytic N_2RR to ammonia	Fe doped TiO_2 nanofibers	0.06	/	8	0.0024	*Angew. Chem. Int. Ed.*, **2021**, *60*, 16085-16092.
Photocatalytic N_2RR to ammonia	Al-PMOF (Fe)	0.007	/	15	0.001	*ACS Nano*, **2021**, *15*, 9670-9678.
Electrocatalytic NO_3^-RR to ammonia	Cu-PTCDA	1.97	85.9	40	0.61	*Nat. Energy*, **2020**, *5*, 605-613.
Electrocatalytic NO_3^-RR to ammonia	Pd nanocrystals	3.611	35.1	4	0.002	*ACS Catal.*, **2021**, *11*, 7568-7577.
Electrocatalytic NO_3^-RR to ammonia	Cu@Th-BPYDC	1500	92.5	/	/	*ACS Cent. Sci.*, **2021**, *7*, 1066-1072.
Electrocatalytic NO_3^-RR to ammonia	BCN@Cu	576.2	88.9	20	2.30	*J. Mater. Chem. A*, **2021**, *DOI: 10.1039/D1TA05718A*.

Electrocatalytic NO_3^-RR to ammonia	Fe SAC	1176	75	35	16.46	*Nat. Commun.*, **2021**, *12*, 2870.
Electrocatalytic NO_3^-RR to ammonia	Cu-NBs-100	655	97%	20	3.28	*Energy Environ. Sci.*, **2021**, *14*, 4989-4997.
Electrocatalytic NO_3^-RR to ammonia	Strained Ru NCs	5560	100	100	102.86	*J. Am. Chem. Soc.*, **2020**, *142*, 7036-7046.
Electrocatalytic N_2RR to ammonia	PdCu-TiO_2	2.92	/	12	0.07	*Nat. Chem.*, **2020**, *12*, 717-724.
Electrocatalytic N_2RR to ammonia	Bismuth nanocrystals	200	66	50	0.52	*Nat. Catal.*, **2019**, *2*, 448-456.
Electrocatalytic N_2RR to ammonia	Ru@ZrO_2/NC	0.26	21	60	0.03	*Chem*, **2019**, *5*, 204-214.
Electrocatalytic N_2RR to ammonia	Fe_{SAs}-MoS_2	36.1	31	50	9.025	*Chem*, **2020**, *6*, 1-17.
Electrocatalytic N_2RR to ammonia	Cu-TiO_2 (OVs)	1.52	21.99	12	0.04	*Adv. Mater.*, **2020**, *32*, 2000299.
Electrocatalytic N_2RR to ammonia	CoPC NTs	6.29	27.7	20	0.126	*ACS Nano*, **2021**, *15*, 5230-5239

Electrocatalytic N_2RR to ammonia	Ru single atoms on N-doped carbon	7.06	29.6	12	0.09	*Adv. Mater.*, **2018**, *30*, 1803498.
Electrocatalytic N_2RR to ammonia	Black phosphorus nanosheets	1.85	5.07	10	0.004	*Angew. Chem. Int. Ed.*, **2019**, *58*, 2612-2616.
Electrocatalytic N_2RR to ammonia	Fe single-atoms on MoS_2 nanosheets	0.51	18.8	12	0.02	*Angew. Chem. Int. Ed.*, **2020**, *59*, 20411-20416.
Electrocatalytic N_2RR to ammonia	$Ti_3C_2T_x$ Mxene nanosheets	0.16	5.78	12	0.0002	*Joule.*, **2019**, *3*, 279-289.
Electrocatalytic N_2RR to ammonia	Pd_{SAs}-Cu NCs	4.07	24.8	30	0.059	*Angew. Chem. Int. Ed.*, **2020**, *60*, 345-350.
Electrocatalytic N_2RR to ammonia	Body-centered cubic PdCu nanoparticles	2.10	11.5	10	0.004	*Angew. Chem. Int. Ed.*, **2020**, *59*, 2649-2653.
Electrocatalytic N_2RR to ammonia	Pt/Au-ZIF	9.47	44	/	/	*Angew. Chem. Int. Ed.*, **2020**, *59*, 16997-17003.
Electrocatalytic N_2RR to ammonia	C doped TiO_2	0.87	17.8	11	0.006	*Angew. Chem. Int. Ed.*, **2019**, *58*, 13101-13106.
Photoelectrochemical N_2RR to ammonia	Aerophilic-hydrophilic Au-Si heterostructures	/	37.8	50	0.007	*Chem*, **2020**, *5*, 617-633.

Photoelectrochemical N_2RR to ammonia	Black phosphorus nanosheets	6.02	23.3	12	0.007	*Adv. Funct. Mater.*, **2020**, *30*, 2002731.
Photoelectrochemical N_2RR to ammonia	Cu_2S-In_2S_3 heterostructure	2.78	33.3	24	0.033	*J. Mater. Chem. A*, **2021**, *9*, 10497-10507.
Photoelectrochemical N_2RR to ammonia	$MoSe_2$-C_3N_4 heterostructure	0.45	28.9	20	0.009	*J. Mater. Chem. A*, **2021**, *9*, 2742-2753.

Supplementary Table 3 Source and purity of chemicals used in this work

Chemicals	**Purity**	**Source**
Hydrofluoric Acid	48%-50%	Adamas
Titanium(IV) butoxide	99%	Adamas
Ethanol	99.7%	Greagent
Potassium nitrate	99.0%	Greagent
Magnesium chloride hexahydrate	99%	Adamas
Calcium chloride hydrate	99.0%	Acros
Strontium chloride hexahydrate	99%	Adamas
Barium chloride dihydrate	99.5%	Greagent
Phenol	≥99.0%	Greagent
Benzyl alcohol	99%	Adamas
Formaldehyde	37.0%-40.0%	Greagent
Cobalt(II) chloride hexahydrate	99%	Adamas
Nickel(II) chloride hexahydrate	99%	Adamas
Cadmium chloride hydrate	98%	Sigma-Aldrich
Potassium sulfate	≥99.0%	Greagent
Potassium phosphate	99%	Adamas
Potassium Carbonate	≥99.0%	Greagent
