# Supplementary material for: Subnanometric alkaline-earth oxide clusters for sustainable nitrate to ammonia photosynthesis
Source: Nat Commun. 2022 Mar 1;13:1098. doi: 10.1038/s41467-022-28740-8 (PMC8888631; doi:10.1038/s41467-022-28740-8)
Supplement: Supplementary file 3 — Description of Additional Supplementary Files [file 41467_2022_28740_MOESM3_ESM.pdf]

Subnanometric alkaline-earth oxide clusters for sustainable nitrate to ammonia photosynthesis

Jieyuan Li,[1,2] Ruimin Chen,[1] Jielin Wang,[1] Ying Zhou,[3] Guidong Yang,[4] Fan Dong[1,2*]

1 Research Center for Environmental and Energy Catalysis, Institute of Fundamental and Frontier Sciences, University of Electronic Science and Technology of China, Chengdu 611731, China.

2 Yangtze Delta Region Institute (Huzhou), University of Electronic Science and Technology of China, Huzhou 313000, China.

3 School of New Energy and Materials, Southwest Petroleum University, Chengdu 610500, China.

4 XJTU-Oxford Joint International Research Laboratory of Catalysis, School of Chemical Engineering and Technology, Xi'an Jiaotong University, Xi'an 710049, China.

* Corresponding author: Fan Dong (dongfan@uestc.edu.cn; dfctbu@126.com)

The Supporting Information files includes the following:

1. Main Supplementary Information
2. Raw Data for Maintext
3. Raw Data for Supplementary Details
4. Complete Author Checklist
5. Complete Editorial Policy Checklist
6. Manuscript with Change Marked
